# In *Kluyveromyces lactis* a Pair of Paralogous Isozymes Catalyze the First Committed Step of Leucine Biosynthesis in Either the Mitochondria or the Cytosol

**DOI:** 10.3389/fmicb.2020.01843

**Published:** 2020-08-04

**Authors:** Beatriz Aguirre-López, Ximena Escalera-Fanjul, Jaqueline Hersch-González, Eréndira Rojas-Ortega, Mohammed El-Hafidi, Mijail Lezama, James González, Michele Maria Bianchi, Geovani López, Dariel Márquez, Claudio Scazzocchio, Lina Riego-Ruiz, Alicia González

**Affiliations:** ^1^Departamento de Bioquímica y Biología Estructural, Instituto de Fisiología Celular, Universidad Nacional Autónoma de México, Mexico City, Mexico; ^2^Theoretical Biophysics, Humboldt-Universität zu Berlin, Berlin, Germany; ^3^Departamento de Biomedicina Cardiovascular, Instituto Nacional de Cardiología Ignacio Chávez, Mexico City, Mexico; ^4^Dipartimento di Biologia e Biotecnologie Charles Darwin, Sapienza Università di Roma, Rome, Italy; ^5^Department of Microbiology, Imperial College London, London, United Kingdom; ^6^Institut de Biologie Intégrative de la Cellule (I2BC), Gif-sur-Yvette, France; ^7^División de Biología Molecular, Instituto Potosino de Investigación Científica y Tecnológica AC, San Luis Potosí, Mexico

**Keywords:** gene duplication, functional diversification, oligomerization, feedback control, subcellular localization

## Abstract

Divergence of paralogous pairs, resulting from gene duplication, plays an important role in the evolution of specialized or novel gene functions. Analysis of selected duplicated pairs has elucidated some of the mechanisms underlying the functional diversification of *Saccharomyces cerevisiae* (*S*. *cerevisiae*) paralogous genes. Similar studies of the orthologous pairs extant in pre-whole genome duplication yeast species, such as *Kluyveromyces lactis* (*K. lactis*) remain to be addressed. The genome of *K. lactis*, an aerobic yeast, includes gene pairs generated by sporadic duplications. The genome of this organism comprises the *KlLEU4* and *KlLEU4BIS* paralogous pair, annotated as putative α-isopropylmalate synthases (α-IPMSs), considered to be the orthologs of the *S. cerevisiae ScLEU4*/*ScLEU9* paralogous genes. The enzymes encoded by the latter two genes are mitochondrially located, differing in their sensitivity to leucine allosteric inhibition resulting in *Sc*Leu4-*Sc*Leu4 and *Sc*Leu4-*Sc*Leu9 sensitive dimers and *Sc*Leu9-*Sc*Leu9 relatively resistant homodimers. Previous work has shown that, in a *Scleu4*Δ mutant, *ScLEU9* expression is increased and assembly of *Sc*Leu9-*Sc*Leu9 leucine resistant homodimers results in loss of feedback regulation of leucine biosynthesis, leading to leucine accumulation and decreased growth rate. Here we report that: (i) *K. lactis* harbors a sporadic gene duplication, comprising the *KlLEU4*, syntenic with *S. cerevisiae ScLEU4* and *ScLEU9*, and the non-syntenic *KlLEU4BIS*, arising from a pre-WGD event. (ii) That both, *KlLEU4* and *KlLEU4BIS* encode leucine sensitive α-IPMSs isozymes, located in the mitochondria (*Kl*Leu4) and the cytosol (*Kl*Leu4BIS), respectively. (iii) That both, *KlLEU4* or *KlLEU4BIS* complement the *Scleu4*Δ *Scleu9*Δ leucine auxotrophic phenotype and revert the enhanced *ScLEU9* transcription observed in a *Scleu4*Δ *ScLEU9* mutant. The *Scleu4*Δ *ScLEU9* growth mutant phenotype is only fully complemented when transformed with the syntenic *KlLEU4* mitochondrial isoform. *KlLEU4* and *KlLEU4BIS* underwent a different diversification pathways than that leading to *ScLEU4*/*ScLEU9*. *KlLEU4* could be considered as the functional ortholog of *ScLEU4*, since its encoded isozyme can complement both the *Scleu4*Δ *Scleu9*Δ leucine auxotrophy and the *Scleu4*Δ *ScLEU9* complex phenotype.

## Introduction

Gene duplication is a source of new or specialized biological functions (Ohno, 1970; Lynch et al., 1991; Force et al., 1999; Kellis et al., 2004). Retained duplicate genes (paralogs) can provide increased dosage of the same product, or may go through a process of sub- or neo-functionalization. In the former, both copies of the gene lose and/or specialize a subset of their ancestral functions, in the latter, at least one of the copies acquires a new function (Ohno, 1970; Lynch et al., 1991; Force et al., 1999).

Paralogous genes can originate from Whole Genome Duplication (WGD) (ohnologs) events or from sporadic duplications known as Small Scale Duplications (SSD) (paralogs). The extant yeast genomes from the *Saccharomyces* and closely related genera arose from a WGD event within the Saccharomycetaceae family (Kellis et al., 2004). More recent work established that this duplication originated from the fusion of two different species genomes (Marcet-Houben and Gabaldón, 2015). The lineage that gave rise to *Kluyveromyces lactis* (*K. lactis*) diverged from the *Saccharomyces cerevisiae* (*S*. *cerevisiae*) lineage before the WGD event (Kellis et al., 2004). Accordingly, this yeast exhibits much less overall genetic redundancy (Conde e Silva et al., 2009), suggesting that the paralogs genes present in *K. lactis* originated from independent SSD events (Dujon et al., 2004; Huerta-Cepas et al., 2014). Subfunctionalization of paralogs pairs can be achieved through various non-exclusive molecular mechanisms such as modifications of the coding sequence leading to: (i) changes in the kinetic parameters (DeLuna et al., 2001; Rojas-Ortega et al., 2018), (ii) differential subcellular localization (Marques et al., 2008), (iii) formation of hetero-oligomeric isozymes with emerging biochemical properties (DeLuna et al., 2001; López et al., 2015), or (iv) modifications of the regulatory region determining differential expression of each copy (Avendaño et al., 2005; Quezada et al., 2008; González et al., 2017).

The analysis of the functional diversification of orthologous gene pairs from *S. cerevisiae* and *K. lactis* could allow addressing the question of whether functional diversification of a given paralogous pair follows either the same or distinct diversification pathways in different organisms. An interesting example of duplicated pairs, present in both *S. cerevisiae* and *K. lactis* is that of α-isopropylmalate synthases (α-IPMSs). The *ScLEU4* (YNL104C) and *ScLEU9* (YOR108W) genes from *S. cerevisiae* form part of a duplicated chromosomal block generated from the WGD event (Wolfe and Shields, 1997), while those present in the *K. lactis KlLEU4* (KLLA0F23529G alias KLLA-ORF403) and *KlLEU4BIS* (KLLLA0D14201G alias KLLA-ORF4593) were originated from an independent gene duplication event prior to the WGD (see below).

**FIGURE 1:**
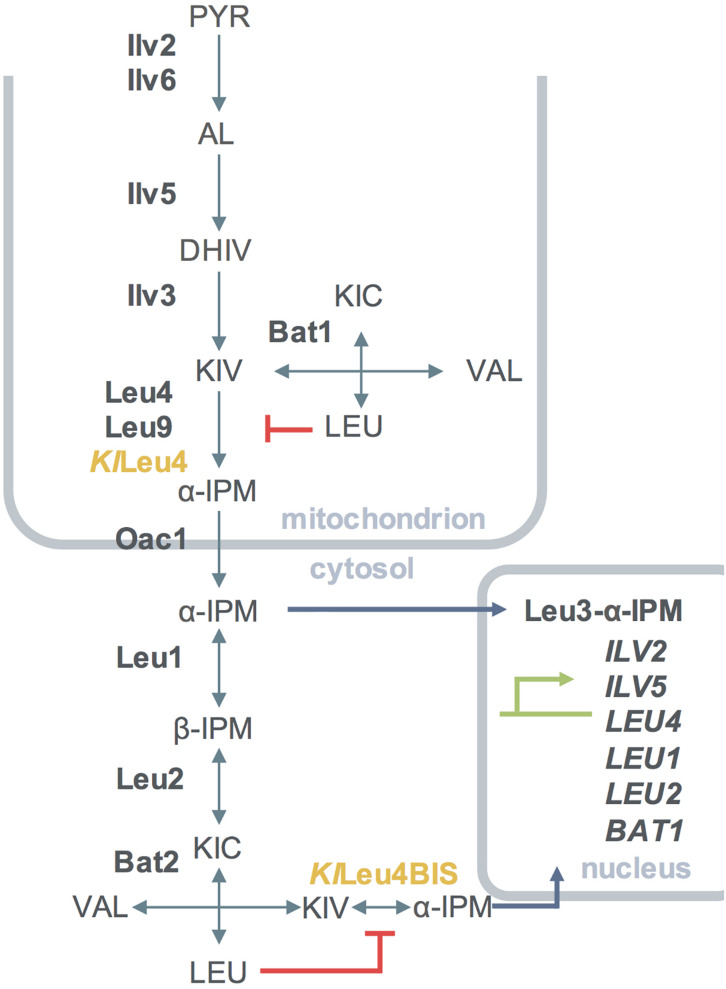
Diagrammatic representation of the compartmentalization of the leucine amino acid biosynthetic pathway of *S. cerevisiae* including *K. lactis* α-IPMSs when transformed in *S. cerevisiae*. Simple and double arrows indicate reactions considered to be irreversible and reversible, respectively. Enzymes from *K. lactis* are shown in yellow, enzymes form *S. cerevisiae* are shown in bold font, metabolites are shown in capital letters, genes activated by Leu3-α-IPM are shown in capital italic letters. Enzymes involved in the pathway are as follows: Ilv2, acetohydroxy acid synthase catalytic subunit; Ilv5, acetohydroxy acid reductoisomerase; Ilv3, dihydroxy acid dehydratase; Bat1, mitochondrial branched-chain amino acid aminotransferase; Bat2, cytosolic branched-chain amino acid aminotransferase; Leu4, α-isopropylmalate synthase, Leu9, α-isopropylmalate synthase; Leu1, isopropylmalate isomerase; Leu2, β-isopropylmalate dehydrogenase, *Kl*Leu4, α-isopropylmalate synthase, *Kl*Leu4BIS, α-isopropylmalate synthase. PYR, pyruvate; AL, acetolactate; DHIV, dihydroxyisovalerate; KIV, ketoisovalerate; α-IPM, α-isopropylmalate; β-IPM, β-isopropylmalate; KIC-α, ketoisocaproate; aCoA, Acetyl coenzyme A.

The *Sc*Leu4 and *Sc*Leu9 α-IPMSs paralogous isozymes from *S. cerevisiae* catalyze the synthesis of α-isopropylmalate (α-IPM) from acetyl coenzyme A (acetyl-CoA) and α-ketoisovalerate (α-KIV), the first committed step of leucine biosynthesis (Ryan et al., 1973; Roeder and Kohlhaw, 1980; Chang et al., 1984, 1985; Beltzer et al., 1988; Cavalieri et al., 1999; Casalone et al., 2000; Kohlhaw, 2003; Figure 1). The catalytic activities of *Sc*Leu4-*Sc*Leu4 and *Sc*Leu9-*Sc*Leu9 homodimers are feed-back inhibited by leucine, albeit with a different kinetics (see below) (Roeder and Kohlhaw, 1980).

*K. lactis* harbors two ORFS with high similarity to *ScLEU4*/*ScLEU9*, *KlLEU4*, and *KlLEU4BIS*, whose physiological role has not been previously analyzed, the successive steps of the leucine biosynthetic pathway being identical to that extant in *S. cerevisiae*. In the latter, the consecutive reactions carried out in the cytoplasm, catalyzed by *Sc*Leu1 and *Sc*Leu2 convert α-IPM to α-ketoisocaproate (α-KIC), which is further converted to leucine by the *Sc*Bat1 and *Sc*Bat2 paralogous aminotransferases (Colón et al., 2011). KLLA0A04961g and KLLA0D04906g are the *K. lactis* orthologous of *ScLEU1* and *ScLEU2*, respectively. Null *Klbat1*Δ mutations result in valine/isoleucine/leucine auxotrophy (Colón et al., 2011). Additionally, the mitochondrial inner membrane α-IPM transporter OacAC1 has also an orthologous counterpart in *K. lactis* (KLLA0B12826g) (Byrne and Wolfe, 2005), however, its functional role has not been studied.

In *S. cerevisiae*, the leucine biosynthesis intermediate α-IPM plays a dual physiological role. On the one hand, it is the leucine precursor (Chang et al., 1985; Kohlhaw, 2003), on the other, it acts as the co-activator of the *Sc*Leu3 master transcriptional regulator (Kohlhaw, 2003), which modulates the expression of a number of genes within and beyond leucine metabolism (Kohlhaw, 2003). ScLeu3 has a dual function (Kohlhaw, 2003). At high α-IPM concentrations it acts as a transcriptional activator of genes involved in branched chain amino acids (Kohlhaw, 2003) and glutamic acid (Hu et al., 1995) biosynthesis. Low intracellular concentrations of α-IPM shift its function to that of a transcriptional repressor of other genes (Kohlhaw, 2003). ORF KLLA0D10593 probably encodes a ScLeu3 *K. lactis* orthologous protein (*Kl*Leu3).

We have previously established how *ScLEU4* and *ScLEU9* have differentially subfunctionalized (López et al., 2015). In *Scleu4*Δ or *Scleu9*Δ single knockout mutants, homodimeric *Sc*Leu4 or *Sc*Leu9 isozymes are present; however, when *ScLEU4* and *ScLEU9* are simultaneously expressed, *Sc*Leu4-*Sc*Leu9 heterodimeric enzymes are preferentially assembled. The *in vivo* assembly of heterodimers occurs since both *Sc*Leu4 and *Sc*Leu9 are located in the mitochondria. The *Sc*Leu4-*Sc*Leu4 isoform is inhibited by low leucine concentrations with an inhibition constant (Kii) of 0.22 mM, while the *Sc*Leu9-*Sc*Leu9 homodimer could be considered as leucine resistant, with a 5.44 mM Kii, thus it is only inhibited by high intracellular concentration of this amino acid. The *Sc*Leu4-*Sc*Leu9 hetero-oligomeric isozyme displays a leucine inhibition constant (Kii 0.70 mM) intermediate to that found for the homo-dimeric isozymes (López et al., 2015).

In a *Scleu4*Δ *ScLEU9* strain, the exclusive presence of the *Sc*Leu9-*Sc*Leu9 leucine-resistant homodimer, results in a growth impaired phenotype, most probably due to the metabolic imbalance produced by the draining of acetyl-CoA to α-IPM and leucine biosynthesis with the consequent depletion of other tricarboxilic acid cycle intermediates (López et al., 2015). This particular phenotype is enhanced by *ScLEU9* overexpression in a *Scleu4*Δ background (López et al., 2015). These results indicate that in *S. cerevisiae*, retention and further diversification of the two α-IPMSs has resulted in a specific regulatory system that controls the leucine-α-IPM biosynthetic pathway through the different feedback sensitivity of homomeric and heterodimeric isoforms. Here we address whether retention and further sub-functionalization of *KlLEU4* and *KlLEU4BIS* paralogs has led to diversification of their physiological roles. We analyze the kinetic properties and sub-cellular localization of the two paralogs and their ability to complement the cognate mutations in *S. cerevisiae*.

## Materials and Methods

### Strain Transformation

*S. cerevisiae* and *K. lactis* strains used in this work are described in Table 1. Yeast strains were transformed following a previously described method (Ito et al., 1983). Transformants were selected for either uracil prototrophy or leucine prototrophy on minimal medium (MM), G418 and/or nourseothricin resistance on yeast extract-peptone-dextrose (YPD)-rich medium and confirmed by PCR.

**TABLE 1:** Strains used in this work.

**Strain**	**Relevant genotype**	**Source**
CLA11-700 CLA1-2	*MATa ScLEU4 ScLEU9 Scleu2*:*ScLEU2 Scura3*	Quezada et al., 2008
CLA11-701 CLA1-2-4	*MATa Scleu4*Δ:*kanMX4 ScLEU9 Scleu2*:*ScLEU2 Scura3*	López et al., 2015
CLA11-702 CLA1-2-4-9	*MATa ScLEU4 Scleu9*Δ:*kanMX4 Scleu2*:*ScLEU2 Scura3*	This study
CLA11-703 CLA1-2-4-9	*MATa Scleu4*Δ:*ScURA3 Scleu9*Δ:*kanMX4 Scleu2*:*ScLEU2*	López et al., 2015
CLA11-701-2 CLA12-4N	*MATa Scleu4*Δ:*natMX4 ScLEU9 Scleu2*:*ScLEU2 Scura3*	This study
CLA11-703-2	*MATa Scleu4*Δ:*natMX4 Scleu9*Δ:*kanMX4 Scleu2*:*ScLEU2 ura3*	This study
CLA11-701-3	*Scleu4*Δ:*KlLEU4-yECitrine ScLEU9 Scleu2*:*ScLEU2 Scura3*	This study
CLA11-701-4	*Scleu4*Δ:*KlLEU4BIS-yECitrine ScLEU9 Scleu2*:*ScLEU2 Scura3*	This study
*K. lactis* CBS2359ku80	*MATa ku80*:*loxP ura3*	Kooistra et al., 2004
*K. lactis* 155-1	*MAT*α *KlLEU4* (*KILAOF23529g*) *KlLEU4BIS* (*KILAOD1421g*) *ade2 his3 ura3*	This study
*K. lactis* 155-2	*MAT*α *Klleu4*Δ:*kanMX4 KlLEU4BIS ade2 his3 ura3*	This study
*K. lactis* 155-3	*MAT*α *KlLEU4 Klleu4BIS*Δ:*kanMX4 ade2 his3 ura3*	This study
*K. lactis* 155-4	*MAT*α *Klleu4*Δ:*natMX4 KlleuBIS*Δ:*kanMX4 ade2 his3 ura3*	López et al., 2015
*K. lactis* CBS2359ku80-1	*MAT*α *KlLEU4-yCitrine KlLEU4BIS ku80*:*loxP ura3*	This study
*K. lactis* CBS2359ku80-2	*MAT*α *KlLEU4 KlLEU4BIS-yCitrine ku80*:*loxP ura3*	This study

### Growth Conditions

*S. cerevisiae* and *K. lactis* strains were pre-grown overnight (16 h) in rich media YPD. Yeast strains were routinely grown on MM containing salts, trace elements, and vitamins following the formula of yeast nitrogen base (Difco). Glucose (2%, w/v) or ethanol (2%, v/v) were used as carbon sources, and 40 mM ammonium sulfate was used as nitrogen source. Supplements needed to satisfy auxotrophic requirements were added at 0.1 mg/mL. Cells were incubated at 30^*o*^C with shaking (250 rpm).

### Construction of *Kluyveromyces lactis Klleu4Δ and Klleu4bisΔ* Mutants

Table 1 describes the genotypes of the strains used in the present work. The single *Klleu4Δ* mutant strain (*K. lactis* 155-2) was constructed as follows: *KlLEU4* (KLLA0F23529g) was replaced by homologous recombination in strain *K. lactis* 155-1 using a module containing the *kanMX4* cassette flanked by 676 bp of 5′ untranslated region (UTR) (−676 to −1) and 474 bp of 3′ UTR (+ 1831 to + 2305) sequences of *KlLEU4*. This module was amplified by overlapped extension PCR with deoxyoligonucleotides A3 and A4 (Supplementary Table S1) using a template built up by three independent modules: (i) the *KlLEU4* 5′ UTR amplified using A3 and A5 deoxyoligonucleotides, (ii) the *kanMX4* module flanked by homologous regions of the 5′ UTR and 3′ UTR of the *KlLEU4* gene, which was amplified from pFA6a plasmid using deoxyoligonucleotides A6 and A7, and (iii) the *KlLEU4* 3′ UTR amplified using deoxyoligonucleotides A8 and A4 (Supplementary Table S1). The PCR product was transformed into the *K. lactis* 155-1 strain. Transformants were selected for G418 resistance. The single *Klleu4bis*Δ mutant strain (*K. lactis* 155-3) was generated as described for *Klleu4Δ*. Three PCR products were amplified as follows: the one corresponding to (i) the 5′ UTR of *KlLEU4BIS* (KLLA0D14201g) was amplified using deoxyoligonucleotides A9 and A10, (ii) the *kanMX4* module flanked by homologous regions to the 5′ and 3′ UTR of *KlLEU4BIS* was amplified from pFA6a plasmid using deoxyoligonucleotides A11 and A12, and (iii) the 3′ UTR of *KlLEU4BIS* amplified using deoxyoligonucleotides A13 and A14, were used as template for an overlapped extension PCR using deoxyoligonucleotides A9 and A14. The assembled PCR product contained the *kanMX4* cassette flanked by 735 bp of 5′ UTR (−735 to −1) and 682 bp of 3′ UTR (+1848 to +2530) of the *KlLEU4BIS* gene. The module was purified and used to transform the *K. lactis* 155-1 strain.

### Construction of *KlLEU4-yECitrine* and *KlLEU4BIS-yECitrine* Tagged Mutants

Deoxyoligonucleotides were designed on the basis of *KlLEU4* A19 and A20 and *KlLEU4BIS* A21 and A22 gene sequence carrying 18 bp of *yECitrine* (forward) or *ScURA3* (reverse) (see Supplementary Table S1) and used to PCR amplify *yECitrine* and *URA3* from plasmid pKT175 (Sheff and Thorn, 2004). The resulting modules were selectively transformed into CBS2359 *ku80Δ* (Kooistra et al., 2004), to generate strains CBS2359 *ku80Δ*-1 (*MATα KlLEU4*-*yECitrine KlLEU4BIS ku80*:*loxP ura3*) and CBS2359 *ku80Δ*-2 (*MATα KlLEU4 KlLEU4BIS*-*yECitrine ku80*:*loxP ura3*) (Table 1).

### Construction of *S. cerevisiae* Strains Harboring *KlLEU4-yECitrine* and *KlLEU4BIS-yECitrine*

To construct *S. cerevisiae* mutant strains harboring either *KlLEU4-yECitrine* or *KlLEU4BIS-yECitrine* (Table 1), we used *Scleu4*Δ:*kanMX4 ScLEU9* (CLA11-701) mutant to replace the *kanMX4* cassette for either the *KlLEU4-yECitrine* or *KlLEU4BIS-yECitrine* generating *Scleu4*Δ:*KlLEU4-yECitrine ScLEU9* (CLA11-701-3) and *Scleu4*Δ:*KlLEU4BIS-yECitrine ScLEU9* (CLA11-701-4) mutant strains. To obtain *Scleu4*Δ:*KlLEU4-yECitrine LEU9*, the *KlLEU4-yECitrine* tagged gene (4409 bp) was PCR amplified from the genomic DNA obtained from the CBS2359 *ku80*Δ-1 strain using the A23 and A24 deoxyoligonucleotide pair (Supplementary Table S1). Using this amplicon as a template, a subsequent PCR was performed using A25 and A26 deoxyoligonucleotides in order to obtain a module (4529 bp) whose 5′ end contains an homologous region with *ScLEU4* promoter from −150 to −91 and in the 3′ end there is an homologous region with 3′ UTR of *ScLEU4* from +1951 to +2010. This module was used to replace *kanMX4* cassette from the strain CLA11-701 (Table 1). Transformants were selected for uracil prototrophy and confirmed by PCR using A27 and A28 deoxyoligonucleotides, the non-recombinants generated a 2330 bp fragment, while in the transformants a 5048 bp fragment was amplified.

Same strategy was followed to obtain *Scleu4*Δ:*KlLEU4BIS-yECitrine ScLEU9*. To amplify *KlLEU4BIS-yECitrine* tagged gene (4468 bp) deoxyoligonucleotides A29 and A30 were used with a genomic DNA obtained from CBS2359 *ku80*Δ-2 strain. Subsequently, a second PCR was carried out using this fragment as a template, for this case primers A31 and A32 were used in order to generate an amplicon (4588 bp) whose 5′ end is homologous to *ScLEU4* promoter from −150 to −91 and the 3′ end is homolog to the *ScLEU4* 3′ UTR from +1951 to +2010. This module was used to replace *kanMX4* cassette from strain CLA11-701. In this case confirmation was carried out by fluorescent microscopy.

### Construction of YEpKD352 Plasmids Harboring *KlLEU4, KlLEU4BIS, ScLEU4*, and *ScLEU9*

Standard molecular biology techniques were followed as previously described (Sambrook et al., 1989). Genes were amplified with their 5′ UTR sequence and cloned into plasmid YEpKD352 (pKD1 or *URA3*). For *KlLEU4*, a 2984 bp region between −933 from the start codon and + 221 from the stop codon was amplified with deoxyoligonucleotides A33 and A34 (Supplementary Table S1). For *KlLEU4BIS*, a 2917 bp region at −1000 bp from the start codon and + 165 from the stop codon was amplified with deoxyoligonucleotides A35 and A36. PCR modules were digested and cloned in order to generate the plasmids YEpKD352-*KlLEU4* and YEpKD352-*KlLEU4BIS*. Genes cloned in YEpKD352 were transformed into strain *K. lactis* 155-4 or in *S. cerevisiae* strains CLA1-2-4 and CLA1-2-4-9. Transformants were selected for either uracil prototrophy or leucine prototrophy on minimal medium (MM).

### Fluorescent Microscopy

To confirm mitochondrial localization of *Kl*Leu4, *Kl*Leu4-yECitrine tagged strain was stained with MitoTracker Red CMXRos (Molecular Probes) according to manufacturer’s specifications. Co-localization between the MitoTracker and yECitrine was determined through sequential imaging. Confocal images were obtained using a FluoView FV1000 laser confocal system (Olympus) attached/interfaced to an Olympus IX81 inverted light microscope with a 60x oil-immersion objective (UPLASAPO 60x O NA:1.35), zoom x20.0 and 3.5 μm of confocal aperture. The excitation and emission settings were as follows: yECitrine excitation at 488 nm; emission 520 nm BF 500 nm range 30 nm; MitoTracker excitation 543 nm; emission 598 nm, BF 555 nm range 100 nm. The subsequent image processing was carried out with Olympus Fluo View FV1000 (version 1.7) software.

### Northern Blot Analysis

Northern blot analysis was carried out as previously described (Struhl and Davis, 1981). Total yeast RNA was prepared from 100 mL aliquots of cultures grown to an optical density at 600 nm (OD_600__*nm*_) ∼0.6 on MM with ammonium sulfate as nitrogen source and glucose (2%, w/v) or ethanol (2%, v/v) as carbon source. PCR products were used as probes, for *KlLEU4* a 1793 bp product was amplified with deoxyoligonucleotides A43 and A44, for *KlLEU4BIS* a 1644 bp product was amplified with deoxyoligonucleotides A45 and A46 (Supplementary Table S1). As internal loading standard, a probe of 477 bp from 18S was PCR amplified with deoxyoligonucleotides A37 and A38. For *ScLEU4*, an 1860 bp product was amplified with deoxyoligonucleotides A39 and A40. For *ScLEU9*, an 1815 bp PCR product was amplified with deoxyoligonucleotides A41 and A42. A 877 bp *ScACT1* fragment amplified with deoxyoligonucleotides A47 and A48 was used as internal loading standard. Blots were scanned using the program ImageQuant 5.2 (Molecular Dynamics).

### *K. lactis*α-IPMSs Purification: *Kl*Leu4 and *Kl*Leu4BIS Overexpression in *E. coli*

For *Kl*Leu4 (KLLA0F23529) heterologous expression, the Bl21 (DE3) *E. coli* strain (Novagen) was transformed. *Kl*Leu4 selected clones were grown in LB medium with 50 mg mL^–1^ of kanamycin, grown at 30^*o*^C with shaking (250 rpm). When cultures reached an OD_600__*nm*_ of 0.4, expression of *KlLEU4* was induced with 400 mmol L^–1^ of isopropyl-β-d-thiogalactopyranoside (IPTG), incubated overnight at 16^*o*^C with shaking (250 rpm), harvested by centrifugation at 1100 g for 15 min, and the cellular pellet was stored at −70^*o*^C until used.

For *Kl*Leu4BIS (KLLA0D14201) heterologous expression, the Rosetta 2TM (DE3) *E. coli* strain (Novagen) was transformed. *Kl*Leu4BIS selected clones were grown in LB medium with 50 mg mL^–1^ of kanamycin and 70 mg mL^–1^ of chloramphenicol, grown at 30^*o*^C with shaking (250 rpm). When cultures reached an OD_600__*nm*_ of 0.6, expression of *Kl*Leu4BIS was induced with 400 mmol L^–1^ of IPTG, incubated overnight at 16^*o*^C with shaking (250 rpm), harvested by centrifugation at 1100 g for 15 min, and the cellular pellet was stored at −70^*o*^C until used.

### Preparation of Whole Cell Soluble Protein Extracts

The cellular pellet of *Kl*Leu4 and *Kl*Leu4BIS strains was suspended in 20 mL of 50 mM K_2_HPO_4_, 0.5 M NaCl, 1 mmol L^–1^ EDTA, 1 mmol L^–1^ dithiothreitol, 1 mmol L^–1^ phenylmethylsulfonyl fluoride (PMSF), pH 8. Soluble extracts were obtained by sonication (Ultrasonic Processor Model: VCX130) with a tip sonicator maintaining the tubes on ice; five cycles (70% amplitude, 1 sec on and 1 sec off for 1 min) with 1 min of incubation on ice between each cycle. After centrifugation at 1100 g for 20 min at 4^*o*^C, the supernatant was stored at 4^*o*^C.

### Immobilized Metal Affinity Chromatography (IMAC)

To purify KlLeu4 protein (KLLA0F23529, syntenic), supernatant was also purified through an equilibrated nickel column (Ni-NTA Agarose, Quiagen), and following the protocol of purification of *Kl*Leu4 with the difference that 2 extra wash steps were added. The first extra wash was 2 volumes of 30 mmol L^–1^ imidazol and the second was 2 volumes of 60 mmol L^–1^ imidazol. To purify *Kl*Leu4BIS protein (KLLA0D14201, non-syntenic), the supernatant was loaded on an equilibrated nickel column (Ni-NTA Agarose, Quiagen), washed with 100 volumes of lysis buffer, 50 volumes of 2 mmol L^–1^ imidazol, 2 volumes of 5 mmol L^–1^ imidazol, 2 volumes of 10 mmol L^–1^ imidazol and 2 volumes of 20 mmol L^–1^ imidazol. The protein was eluted with 2 volumes of 50, 100, 200, and 300 mmol L^–1^ imidazol and stored at 4^*o*^C until used. *Kl*Leu4 and *Kl*Leu4BIS homogeneity was verified by denaturing with a polyacrylamide gel electrophoresis (12%, SDS-PAGE) and the gel stained with Coomassie Blue. Proteins were 10-fold concentrated with AmiconR Ultra-15 10K Centrifugal Filter Devices (Millipore), and then diluted to the original sample volume with assay buffer (50 mM K_2_HPO_4_, pH 7.5) three “washing out” cycles were performed.

### α-Isopropylmalate Synthase Enzyme Assay and Protein Determination

Cells were grown to an OD_600__*nm*_ ∼0.6 on glucose (2%, w/v) or ethanol (2%, v/v) as carbon sources, and samples were collected at this point. *S. cerevisiae* crude extracts were obtained by disrupting the cells with glass beads in phosphate buffer A (50 mM phosphate buffer pH 7.5, 1 mM PMSF and 1 mM DTT). For *K. lactis* strains, lysis buffer was prepared with 50 mM HEPES buffer pH 7.5 and protease inhibitor cocktail tablets (Roche, cat. No. 11697 498001). The α-isopropylmalate synthase activity was assayed with 5 mM 5,5′-dithiobis-2-nitrobenzoic acid (DTNB, Ellman’s reagent) in 1.0 mL of medium containing 50 mM phosphate buffer pH 7.5, 140 mM KCl, 4 mM MgCl_2_, 0.25 mM acetyl coenzyme A (acetyl-CoA) and 10 mM α-ketoisovalerate (α-KIV). After 5 min of thermal equilibration at 30°C and completion of the reaction of DTNB with the contaminant CoA present in the commercial acetyl-CoA preparation, assay was started by adding cell free extract and the initial reaction rate was obtained from the absorbance change at 412 nm in a Varian Cary 400 spectrophotometer with a 1 cm path length. The 2-nitro-5-thiobenzoic acid (NTB^2–^) production was quantified using an extinction coefficient of 14.15 M^–1^ cm^–1^ (Riddles et al., 1983). Specific activity is given in nmoles of CoA formed per minute. Protein concentration was determined as described previously (Lowry et al., 1951).

### Enzyme Kinetics and Data Analysis

Initial velocity measurements were performed varying both substrates (for *Kl*Leu4, 0.002–0.1 mM acetyl-CoA and 0.002–0.08 mM α-KIV. While for *Kl*Leu4BIS, 0.008–0.1 mM acetyl-CoA and 0.01–0.25 mM α-KIV) and results were globally fitted to equation 1 (Eq. 1) using the Prism software. The resulting kinetic parameters are shown in Table 2.

**TABLE 2:** *Kl*Leu4 and *Kl*Leu4BIS fitted kinetic parameters.

	***Vmax***	***Ka***	***Kia***	***Kb***	***Kis***	***Kii***
	
	**(mMol min^–1^ mg^–1^)**	**(mM)**				
*Kl*Leu4	3.864 ± 0.147	0.026 ± 0.003	0.017 ± 0.005	0.025 ± 0.003	0.034 ± 0.011	0.073 ± 0.012
*Kl*Leu4BIS	2.580 ± 0.241	0.019 ± 0.005	0.012 ± 0.007	0.055 ± 0.013	0.031 ± 0.008	0.090 ± 0.015

**Values represent the fitted kinetic parameters ± standard error. For all the fitting analysis the R^2^ was higher than 0.976. Similar Kis and Kii values were obtained using Eqs 2 or 3 as described in section “Materials and Methods” section.**

(1) $$v=\frac{V_{m\,a\,x}\,\left[A\right]\,\left[B\right]}{K\,i\,a\,K\,b+K\,b\,\left[A\right]+K\,a\,\left[B\right]+\left[A\right]\,\left[B\right]}$$

Where *v*, represents initial velocity; *Vmax*, maximal velocity; *A*, acetyl-CoA concentration; *B*, α-KIV concentration; *Ka* and *Kb* are the *Km* values for *A* and *B* respectively and *Kia* is the dissociation constant for *A*. To study leucine inhibition, saturation curves were performed at different leucine concentrations: 0, 0.01, 0.02, 0.06 and 0.12 for both enzymes. At each leucine concentration, acetyl-CoA was varied (0.002–0.06 mM) while α-KIV was fixed to 0.25 mM. When α-KIV was varied (0.005–0.1 mM), acetyl-CoA concentration was fixed to 0.1 mM, the double reciprocal plot corresponded to a mixed inhibition model. Results were globally fitted using the Prism software to Eq. 2 when the varied substrate was acetyl-CoA, and to Eq. 3 when **α** −KIV was the variable substrate. In Eqs 2 and 3: *I*, leucine concentration; *Kis* is the slope associated inhibitor constant which reflects the extent of leucine dissociation to the free enzyme and *Kii* is the intercept associated inhibitor constant which indicates the extent of leucine dissociation to the enzyme-substrates complex. The other parameters have the same meaning as in Eq. 1. The resulting kinetic parameters are shown in Table 2.

$$v=\frac{V_{\text{max}}\,\left[A\right]}{K\,a\,\left(1+\frac{K\,i\,b}{B}\right)\,\left(1+\frac{I}{K\,i\,s}\right)+\left[A\right]\,\left(1+\frac{K\,b}{B}\right)\,\left(1+\frac{I}{K\,i\,i}\right)}$$

(2) $$v=\frac{V_{m\,a\,x}\,\left[B\right]}{K\,b\,\left(1+\frac{K\,i\,a}{A}\right)\,\left(1+\frac{I}{K\,i\,s}\right)+\left[B\right]\,\left(1+\frac{K\,a}{A}\right)\,\left(1+\frac{I}{K\,i\,i}\right)}$$

### Metabolite Extraction and Analysis

Cell extracts were prepared from exponentially growing cultures. Samples used for intracellular amino acid determination were treated as previously described (Quezada et al., 2008).

### Data Analysis

Statistical analysis was performed using GraphPad Prism software version 8.0, for OS X. For each condition, the wild type *KlLEU4 KlLEU4BIS* strain was considered as control and one-way Analysis of Variance (ANOVA) tests was performed. Significant differences were evaluated by the Dunnett’s multiple comparisons test. Results were considered significant at *p* < 0.05.

### *In silico* and Bioinformatic Methods

**FIGURE 8:**
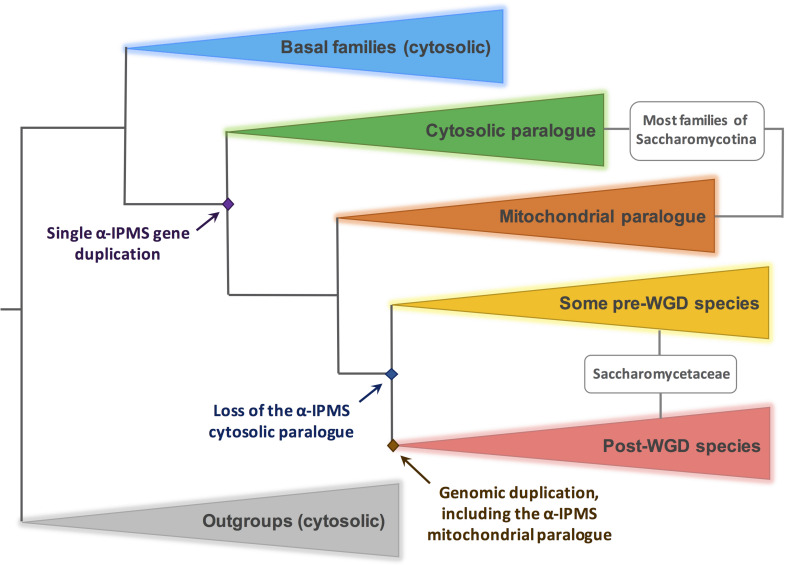
Duplication and loss events of genes encoding α-IPMS in the Saccharomycotina. A simplified scheme, based on the results of a comprehensive phylogeny shown in Supplementary Figure S2. The outgroup (gray triangle) include α-IPMS genes of representative species of Taphrinomycotina and Pezizomycotina. In these clades only one paralog is extant, presumably cytosolic (see text and Supplementary Data Sheet 1). The blue triangle includes basal species of Saccharomycotina where no α-IPMS gene duplication has occurred. Within the Saccharomycotina, a gene duplication occurred (green and brown triangles), leading to the genomes of most species comprising two isoforms, presumably cytosolic and mitochondrial respectively. *KlLEU4BIS* and *KlLEU4* are included in the green and brown clade respectively. In several pre-WGD members of the Saccharomycetacea (represented by a yellow triangle), including those giving origin to the WGD, the cytosolic paralog was lost. Thus, the WGD results in two mitochondrial isoforms (red triangle). *ScLEU4* and *ScLEU9* originate from this duplication.

The following methods were used in the construction of Figure 8 and Supplementary Figures S2, S3. Peptidic sequences of putative orthologs of the *S. cerevisiae Sc*Leu4, *Sc*Leu9, and *K. lactis Kl*Leu4 and *Kl*Leu4BIS were obtained by BlastP from the databases of NCBI and JGI (Mycocosm). All publicly available genomes of the Saccharomycotina were mined. We included orthologs from representative members of the Taphrinomycotina and Pezizomycotina as outgroups. Alignment was carried out with MAFFT G-INS-I with default parameters^1^ (Kuraku et al., 2013; Katoh et al., 2019). Sequences were trimmed with trimAl, a tool for automated alignment trimming in large-scale phylogenetic analysis (Capella-Gutiérrez et al., 2009). A Maximum Likelihood tree was constructed with PhyML^2^ (Guindon et al., 2005). Statistical test for branch support is indicated by Approximate Likelihood Ratio test (aLTR, SH-like) (Anisimova and Gascuel, 2006). The tree was visualized and edited with FigTree v1.4.3.^3^. Putative location (cytosolic or mitochondrial) was predicted for each sequence with Mitoprot^4^ (Claros and Vincens, 1996) and BacelLo^5^ (Pierleoni et al., 2006). After the tree was completed the putative localization of each protein was checked with DeepLoc-1.0^6^ (Almagro Armenteros et al., 2017). The number of duplicated paralogous pairs occurring in *K. lactis* in relation to other species was determined using MetaPhOrs^7^ (Chorostecki et al., 2020).

## Results

### *KlLEU4*/*KlLEU4BIS* Encode α-Isopropylmalate Synthases

As mentioned above, the *K. lactis* genome sequence, indicates the existence of two paralogs genes presumably encoding α-isopropylmalate synthases (α-IPMSs): *KlLEU4* (syntenic with *ScLEU4*/*ScLEU9*) and *KlLEU4BIS* (non-syntenic), respectively located on chromosome XIV and XV.

**FIGURE 2:**
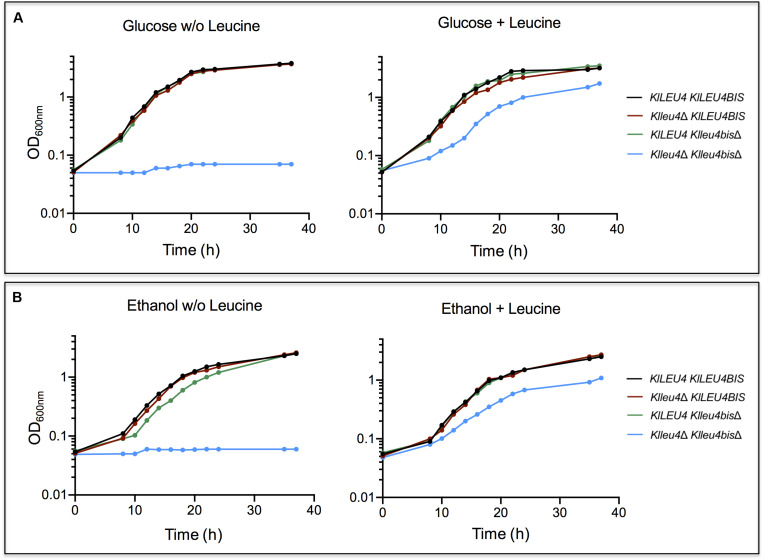
*K. lactis* growth curves of derived single and double *KlLEU4* and *KlLEU4BIS* mutants. Strains were grown on minimal medium with 2% w/v glucose **(A)** or 2% v/v ethanol **(B)** as carbon sources, with or w/o leucine (0.1 mg/mL). Growth curves of wild type *KlLEU4 KlLEU4BIS* strain are shown as growth controls. Experiments were done in duplicate.

To analyze the biological function of the two paralogs genes from *K. lactis*, deletion mutants in each one of the two genes (*Klleu4*Δ and *Klleu4bis*Δ) were constructed (section “Materials and Methods” and Table 1). Single null mutations in either one of the two genes showed wild type growth rate in cultures supplemented with ammonium as nitrogen source and glucose (Figure 2A) or ethanol (Figure 2B) as sole carbon sources. Only the double *Klleu4*Δ *Klleu4bis*Δ mutant displayed leucine auxotrophy (Figures 2A,B). The addition of leucine to the media did not fully restore its growth rate to the wild type level (either on glucose or on ethanol) (Figures 2A,B), suggesting that simultaneous loss of *KlLEU4* and *KlLEU4BIS* could result in an additional requirement(s) not fulfilled by the sole addition of leucine. This will be addressed below (see Discussion).

**FIGURE 3:**
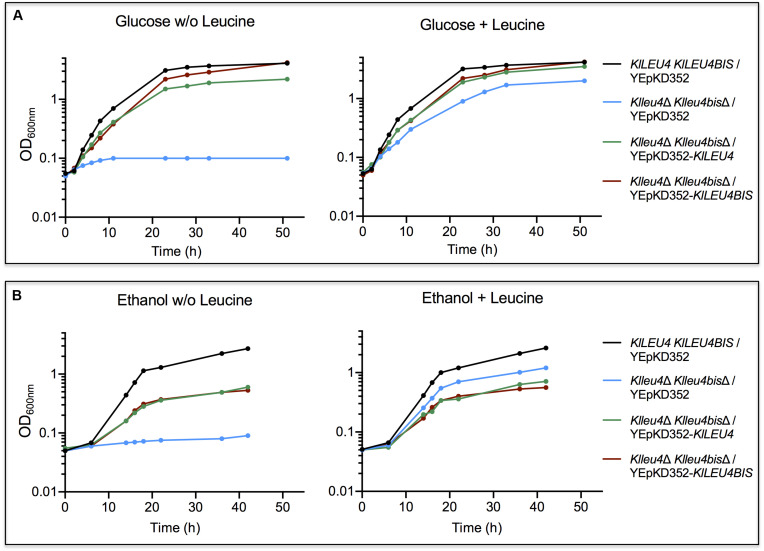
*K. lactis* growth curves of double *Klleu4*Δ *Klleu4bis*Δ mutant transformed with *KlLEU4* or *KlLEU4BIS*. Strains were grown on minimal medium with either 2% w/v glucose **(A)** or 2% v/v ethanol **(B)** as carbon sources, with or w/o leucine (0.1 mg/mL). Growth curves of wild type *KlLEU4 KlLEU4BIS* harboring YEpKD352 are shown as growth controls. Experiments were done in duplicate.

As expected, it was observed that when grown on glucose, leucine auxotrophy displayed by the *Klleu4*Δ *Klleu4bis*Δ strain was complemented with plasmids harboring either one of the *K. lactis* paralogous genes (Figure 3A). However, when grown on ethanol, the double mutant transformed with plasmids harboring either *KlLEU4* or *KlLEU4BIS*, did not fully recover the wild type phenotype, not even in the presence of leucine (Figure 3B). The *K. lactis* genes were cloned in a multicopy plasmid which could result in increased α-IPMS activity, and thus in a metabolic imbalance due to the draining of acetyl-CoA to α-IPM and leucine biosynthesis depleting other TCA cycle intermediates, and consequently decreasing growth rate (López et al., 2015). Although, in glucose we also see this higher activity the TCA cycle is more relevant in ethanol.

As expected, in the presence of the empty vector, leucine addition allowed growth recovery, although it did not reach wild type rate, supporting the proposal that leucine does not fulfill the need of a not yet identified, additional requirement (Figure 3B).

**FIGURE 4:**
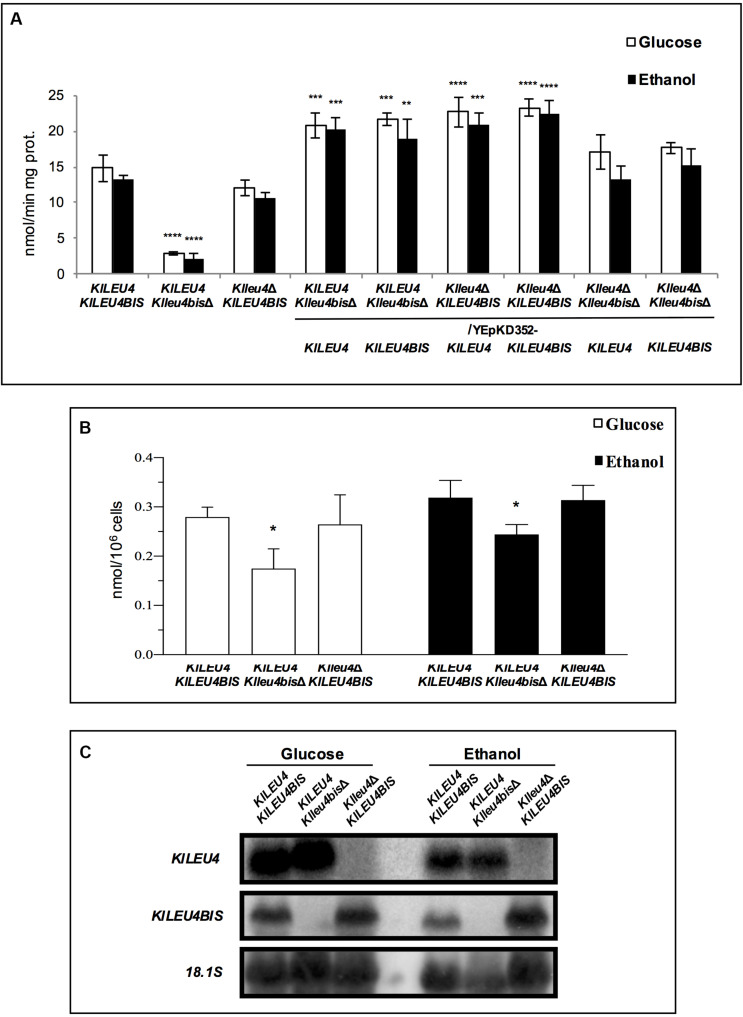
α-IPMS activity, leucine pools and expression profiles determined in extracts obtained from cultures of *K. lactis* mutant strains. **(A)** Enzymatic activity of derived single and double *Klleu4*Δ and *Klleu4bis*Δ mutants transformed with *KlLEU4* or *KlLEU4BIS.* Wild type *KlLEU4 KlLEU4BIS* and derived single *KlLEU4* and *KlLEU4BIS* mutants were used as control. Extracts were prepared from cells grown on minimal medium with either 2% w/v glucose or 2% v/v ethanol as sole carbon sources. **(B)** Leucine pools of wild type *KlLEU4 KlLEU4BIS* and derived single *Klleu4*Δ and *Klleu4bis*Δ mutants were prepared as described in section “Materials and Methods.” In **(A,B)**, bars indicate mean ± SD (*n* = 3), *p < 0.05, **p < 0.01, ***p < 0.001, ***p < 0.0001. **(C)** Northern analysis was carried out as described in section “Materials and Methods.” Total Yeast RNA was prepared from 100 mL aliquots of cultures grown to an OD_600__*nm*_ ∼0.6 on minimal media with 2% w/v glucose or 2% v/v ethanol as carbon sources. Specific probes are described in section “Materials and Methods.” Blots were scanned using the program ImageQuant 5.2 (Molecular Dynamics).

Enzymatic activity of α-IPMS was determined in extracts prepared from glucose or ethanol grown cultures of the wild type strain and *Klleu4*Δ and *Klleu4bis*Δ single mutants. It was found that the *Klleu4*Δ and *Klleu4bis*Δ single mutant strains, retained 80 or 20% of the activity found in a wild type strain, respectively. As expected, the activity fostered by the enzymes encoded in the genes cloned in the multicopy plasmids, was higher than that found in the single mutant strains (Figure 4A). In agreement with *Kl*Leu4 and *Kl*Leu4BIS catalytic contribution, the leucine intracellular pools were higher in the *Klleu4*Δ mutant as compared to that observed in the *Klleu4bis*Δ mutant (Figure 4B).

Previous results showed that *ScLEU4* expression is influenced by the nature of the carbon source (López et al., 2015). Thus, we analyzed whether a similar effect could be observed for *KlLEU4* and *KlLEU4BIS*. Therefore, total RNA preparations were obtained from wild type *K. lactis* glucose or ethanol-grown cultures. Figure 4C shows that both genes have similar expression on either carbon source. We also analyzed the expression profile of each gene when its paralogous was deleted. It was found that absence of either one of the two paralogous genes did not affect expression of the remaining one (Figure 4C).

### *K. lactis* Isozymes Are Located in Different Subcellular Compartments

**FIGURE 5:**
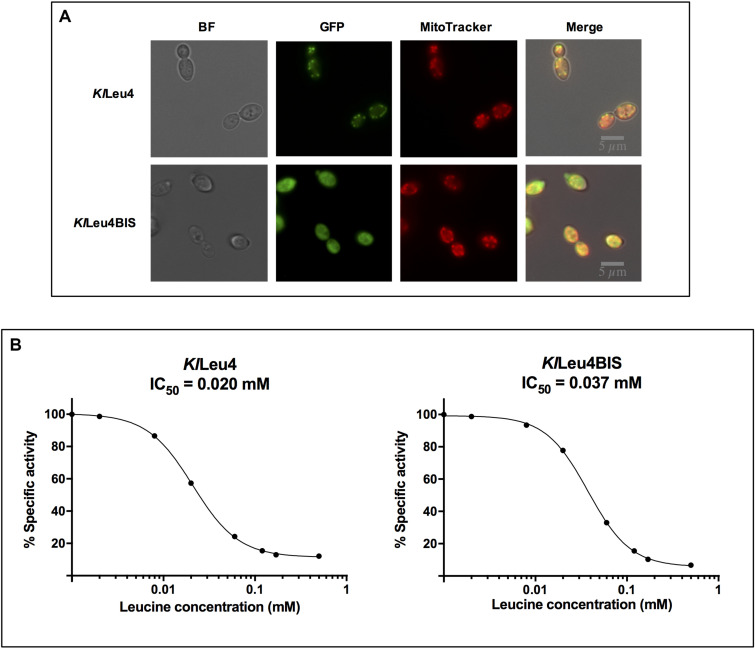
*Kl*Leu4 and *Kl*Leu4BIS isozymes are leucine sensitive and respectively located in mitochondria and cytosol. **(A)** Isozyme leucine sensitivity was carried out on crude extracts as described in section “Materials and Methods.” Experiments were done in duplicate. **(B)** Subcellular localization of *Kl*Leu4 and *Kl*Leu4BIS paralogous proteins tagged with yECitrine using confocal microscopy. Strains were grown on minimal medium glucose (2%, w/v) and collected during exponential phase. Representative image of multiple cells from two biological replicates.

The phylogeny of the α-IPMS encoding-genes, discussed below and in Supplementary Material, strongly suggested the *Kl*Leu4 paralog to be mitochondrial, and *Kl*Leu4BIS to be cytosolic. Figure 5A shows this the case. Thus, it is extremely unlikely that hetero-oligomeric isoforms could form “*in vivo*,” suggesting that the enzymatic activity detected in a wild type strain results from the addition of the independent activity of the *Kl*Leu4 and *Kl*Leu4BIS homodimeric isozymes.

### *Kl*Leu4 and *Kl*Leu4BIS Are Leucine Sensitive

Leucine sensitivity of the two α-isopropylmalate synthases paralogs of *K. lactis* was analyzed. Half-maximal inhibitory concentration (IC_50_) of *Kl*Leu4 and *Kl*Leu4BIS was determined in whole cell extracts obtained from either *Klleu4*Δ or *Klleu4bis*Δ single mutants. As Figure 5B shows, the two *K. lactis* paralogs isozymes showed similar leucine sensitivity: *Kl*Leu4 0.020 mM and *Kl*Leu4BIS 0.037 mM.

The kinetic properties of the purified enzymes (see section “Materials and Methods”) were analyzed (Supplementary Figure S1). Experimental data were fitted to Eq. 1, which corresponds to a compulsory ordered bi-bi reaction under steady state treatment (Segel, 1993). The kinetic properties of the enzymes were similar (Table 2). For both enzymes leucine behaved as a mixed inhibitor, decreasing both, the apparent affinity for the substrates and the apparent maximum enzyme reaction rate (*Vmax*). Experimental data were fitted to Eq. 2 when the acetyl-CoA concentration was varied and to Eq. 3 when the variable substrate was α-KIV (Segel, 1993) (Table 2) (see text and equations in section “Materials and Methods”). This model predicts that leucine can bind for both isoforms either to the free enzyme or to the substrate-bound enzyme. The former process is reflected in the *Kis* constant whereas the latter is reflected in the *Kii* value (Table 2, Eqs 2 and 3 and Figure 5B). From these kinetic parameters we can thus conclude that both enzymes contribute very similarly to the control of the metabolic flux, at variance to the situation in *S. cerevisiae* in which the Leu9 homomeric enzyme showed a *Kii* value which was 24- and 20-fold higher than the one found for the Leu4 homodimer and the Leu4-Leu9 heterodimer, respectively.

As single mutants showed wild type growth phenotypes (see above, Figure 2), *Kl*Leu4 and *Kl*Leu4BIS, although located in different subcellular compartments, must have a redundant role in leucine biosynthesis under the conditions tested.

### Heterologous Complementation of *S. cerevisiae* Double (*Scleu4*Δ *Scleu9*Δ) and Single (*Scleu4*Δ *ScLEU9*) Mutants

**FIGURE 6:**
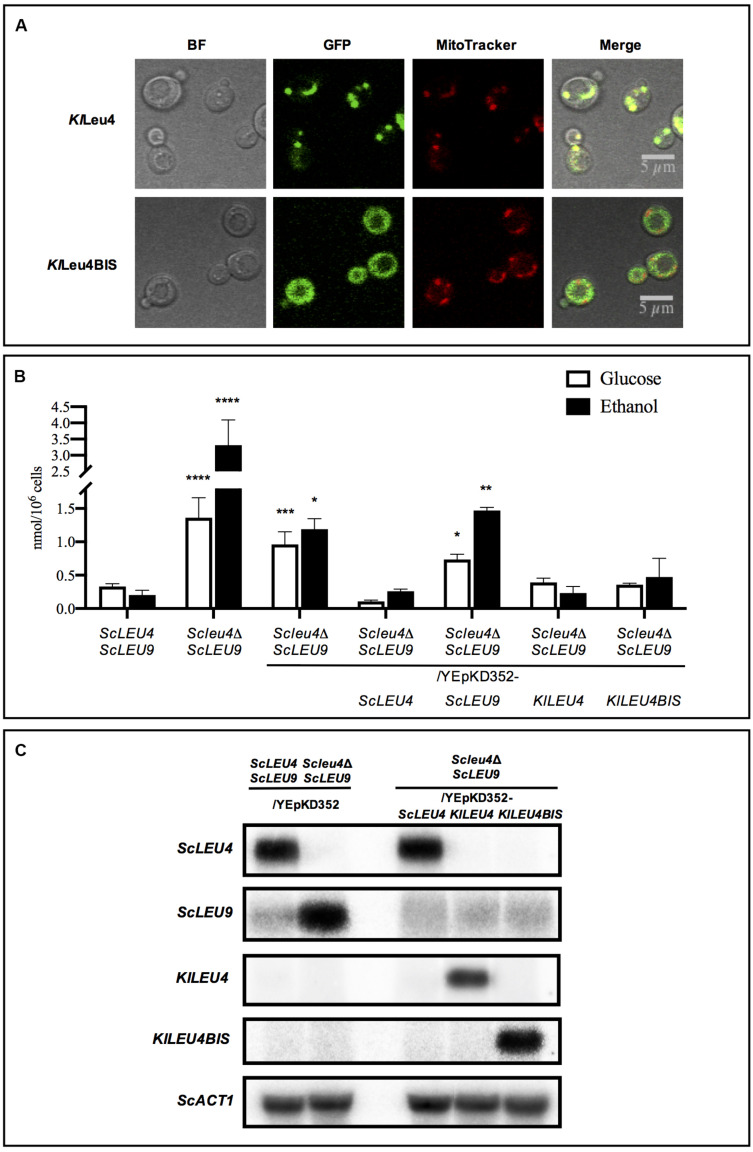
Subcellular localization of *Kl*Leu4 and *Kl*Leu4BIS in *S. cerevisiae*, leucine pools and expression profiles of *Scleu4*Δ *ScLEU9* transformed with *KlLEU4* and *KlLEU4BIS*. **(A)** Subcellular localization in *S. cerevisiae* of *Kl*Leu4 and *Kl*Leu4BIS paralogous proteins tagged with yECitrine using confocal microscopy. Strains were grown on minimal medium glucose (2%, w/v), and collected during exponential phase. Representative image of multiple cells from two biological replicates. **(B)** Leucine pools of wild type *ScLEU4 ScLEU9* and derived single *ScLEU4* mutant harboring plasmids YEpKD352-*ScLEU4*, YEpKD352-*ScLEU9*, YEpKD352-*KlLEU4*, or YEpKD352-*KlLEU4BIS* were prepared as described in section “Materials and Methods. Bars indicate mean ± SD (*n* = 3), ^∗^*p* < 0.05, ^∗∗^*p* < 0.01, ^∗∗∗^*p* < 0.001, ^****^p < 0.0001. **(C)** Northern analysis was carried out as described in section “Materials and Methods.” Total Yeast RNA was prepared from 100 mL aliquots of cultures grown to an OD_600__*nm*_ ∼0.6 on minimal media with 2% w/v glucose as carbon source. Probes used to monitor expression for each gene are described in section “Materials and Methods.” Blots were scanned using the program ImageQuant 5.2 (Molecular Dynamics).

Subcellular localization of the *K. lactis* orthologous isozymes heterologously expressed in *S. cerevisiae* was examined. As shown in Figure 6A, in *S cerevisiae*, both enzymes maintained their native localization: *Kl*Leu4-mitochondrial and *Kl*Leu4BIS-cytosolic. In an *Scleu4*Δ *ScLEU9* strain transformed with the *Kl*Leu4 mitochondrial isoform, *Kl*Leu4-*Sc*Leu9 heterodimers could presumably be formed, thus decreasing *Sc*Leu9-*Sc*Leu9 homodimer concentration, hence counteracting the negative effect on growth of the *Sc*Leu9-*Sc*Leu9 leucine resistant isoform.

**FIGURE 7:**
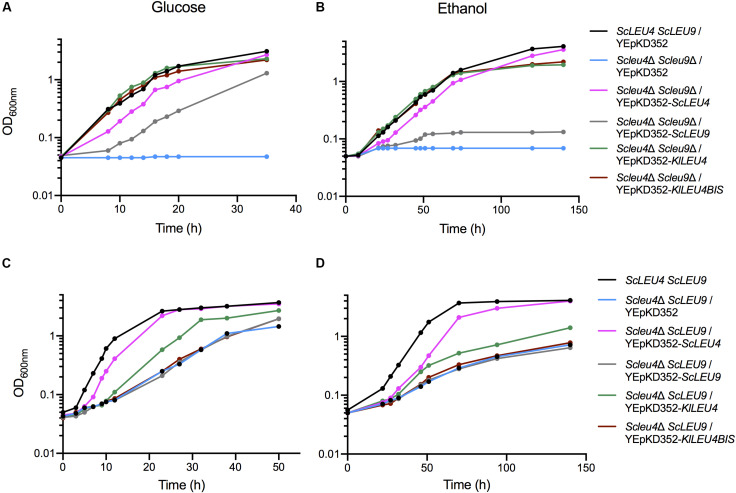
*KlLEU4* and *KlLEU4BIS S. cerevisiae* double and single mutant complementation. Growth curves of *S. cerevisiae Scleu4*Δ *ScLEU9* harboring plasmids YEpKD352, YEpKD352-*ScLEU4*, YEpKD352-*ScLEU9*, YEpKD352-*KlLEU4*, or YEpKD352-*KlLEU4BIS*. Cells were grown on minimal medium with either **(A)** (2%, w/v) glucose, or **(B)** (2% v/v) ethanol. Glucose growth curves of *S. cerevisiae* wild type *ScLEU4 ScLEU9* strain and double *Scleu4*Δ *Scleu9*Δ mutant transformed with YEpKD352-*ScLEU4*, YEpKD352-*ScLEU9*, YEpKD352-*KlLEU4*, or YEpKD352-*KlLEU4BIS*. Strains were grown on minimal medium with **(C)** (2% w/v) glucose or **(D)** (2% v/v) ethanol. Experiments were done in duplicate.

Double *Scleu4*Δ *Scleu9*Δ or single *Scleu4*Δ *ScLEU9* or mutants were independently transformed with YEpKD352 plasmids each harboring *KlLEU4* or *KlLEU4BIS* with their endogenous promoters. As controls, *S. cerevisiae* mutants were also transformed with *ScLEU4* and *ScLEU9* cloned in YEpKD352. We found that, as previously reported (López et al., 2015), when grown on glucose or ethanol, *Scleu4*Δ *Scleu9*Δ transformed with *ScLEU4* grew as well as the wild type strain. However, double mutants transformed with *ScLEU9* were only partially complemented in glucose and not complemented in ethanol (Figures 7A,B). The *Scleu4*Δ *ScLEU9* single mutant strain, when transformed with *ScLEU4* recovered a wild type phenotype, while transformants obtained with *ScLEU9* showed a similar phenotype to that displayed when transformed with the empty YEpKD352 vector (Figures 7C,D). A vector carrying *KlLEU4* partially complemented the phenotype of an *Scleu4*Δ *ScLEU9* strain, while one carrying *KlLEU4BIS* did not complement at all (Figures 7C,D).

Leucine pools were determined in the wild type strain (*ScLEU4 ScLEU9*), *Scleu4*Δ *ScLEU9*, and *Scleu4*Δ *LEU9* transformed with YEpKD352 empty vector or YEpKD352-*ScLEU4, ScLEU9, KlLEU4*, or *KlLEU4BIS*. As previously reported, the *Scleu4*Δ *ScLEU9* mutant accumulated a several-fold higher leucine pool, in either glucose or ethanol, as compared to that found in the wild type strain (Figure 6B). This accumulation was reverted in *ScLEU4* transformants, but not in *ScLEU9* transformants (López et al., 2015; Figure 6B). Surprisingly, although *KlLEU4* but not *KlLEU4BIS* improved the growth rate of the *Scleu4*Δ *ScLEU9* strain, both *KlLEU4* and *KlLEU4BIS* transformants showed intermediate leucine accumulation, ranging between the one observed in the *Scleu4*Δ *ScLEU9* mutant and that found in the *Scleu4*Δ *ScLEU9* transformed with *ScLEU4* (Figure 6B). In an *Scleu4*Δ *ScLEU9* strain, *LEU9* expression is several-fold enhanced (Figure 6C) this together with the leucine insensitivity of *Sc*Leu9-*Sc*Leu9 homodimers would account for the increased leucine pool (López et al., 2015).

### *Scleu4*Δ *ScLEU9*-Dependent Induced *ScLEU9* Over-Expression Is Suppressed in Transformants Harboring *ScLEU4, KlLEU4*, or *KlLEU4BIS*

Northern blot analysis showed that the intermediate leucine pools observed in the *Scleu4*Δ *ScLEU9*, transformed with YEpKD352-*ScLEU4*, YEpKD352-*KlLEU4*, or YEpKD352-*KlLEU4BIS* could be attributed to diminished *ScLEU9* expression, as compared to that found in the *Scleu4*Δ *ScLEU9/*YEpKD352 (Figure 6C). Both the *K. lactis* genes mimic the *ScLEU4* repressive effect on *ScLEU9* expression by hitherto unidentified mechanisms. In a *Scleu4*Δ *ScLEU9* mutant, the reduced leucine sensitivity of *Sc*Leu9-*Sc*Leu9 homodimers would result in increased flux through the leucine biosynthetic pathway, depleting the pool of metabolic intermediates, leading to reduced growth. In the *Scleu4*Δ *ScLEU9*/*KlLEU4* transformed strain, *ScLEU9* expression is decreased, and the reduced growth phenotype is partially alleviated. Additionally, the possible formation of a *Kl*Leu4-*Sc*Leu9 leucine sensitive heterodimer could also contribute to the alleviated growth phenotype. Transformants obtained with *KlLEU4BIS*, encoding the cytosolic isoform, which is presumably unable to form heterodimers with *ScLEU9*, are not complemented for the impaired growth phenotype although *ScLEU9* expression is diminished (Figures 6C, 7C,D).

### Phylogeny of α-Isopropyl Malate Synthase in the Saccharomycotina

Isopropyl malate synthase (α-IPMS) is conserved throughout the tree of life (with the notable exception of metazoans) and it is present in most fungal taxa. The two mitochondrial paralogs of *S. cerevisiae* analyzed in our previous publication (López et al., 2015) originated from the WGD event, which is at the root of the sub-family of Saccharomycetaceae that includes *S. cerevisiae* (Wolfe and Shields, 1997; Marcet-Houben and Gabaldón, 2015; see below).

In this paper, we describe two paralogous *K. lactis* genes encoding leucine sensitive α-IPMSs which differ in their cellular localization. The genes encoding these two isoforms must have originated by a single gene duplication preceding the WGD. We have attempted to pinpoint the origin of this duplication by constructing a phylogeny of α-IPMSs within the Saccharomycotina. A maximum likelihood tree, including as out-groups examples of other ascomycotina (Taprhinomycotina and Pezizomycotina) is shown in Supplementary Figure S2. We have investigated the presumed location of each protein in the tree by three independent algorithms, which detect the presence/absence of a putative mitochondrial entry pre-sequence. The results are described in detail in Supplementary Data Sheet 1 and in the legend to Supplementary Figure S2 and will be briefly summarized below. Figure 8 shows schematically the crucial events of α-IPMSs evolution in Saccharomycotina. The α-IPMSs of Pezizomycotina (such as many plant pathogens and the model organisms *Neurospora crassa* and *Aspergillus nidulans*) and Taphrinomycotina (such as *Schizosaccharomyces pombe*) are presumably cytosolic (see Supplementary Data Sheet 1 for details).

Basal species of Saccharomycotina (such as *Yarrowia lipolytica*) include only one paralog, presumably cytosolic (PCP). A duplication, resulting in a putative mitochondrial isoform (PMP) occurred in coincidence to the emergence of the CUG-Ala clade, a monophyletic, newly classified clade of the Saccharomycotina (Shen et al., 2018, see Supplementary Data Sheet 1). The emergence of the mitochondrial isoform (PMP) occurred presumably through the inclusion of an N-terminal pre-sequence, as exemplified in Supplementary Figure S3. Most families of the Saccharomycotina include species with these two isoforms. Both *S. cerevisiae* and *K. lactis* are members of the *Saccharomycetaceae.* Within this family several events of loss of the presumably cytosolic paralog have occurred (Supplementary Figure S2). The WGD arose seemingly from the hybridization of two distinct species of *Saccharomycetaceae*, one belonging to the *KLE* (*Kluyveromyces*, *Lachancea*, and *Eremothecium) clade, the other to the ZT (Zygosaccharomyces* and *Torulaspora) clade* (Marcet-Houben and Gabaldón, 2015). In both clades, episodes of loss of the cytosolic paralog have occurred (Supplementary Figure S2, noticeably in *Lachancea* species and *T. delbrueckii*), thus resulting in post-WGD species including two mitochondrial isoforms but no cytoplasmic isoforms, not withstanding that further loss of one of the two mitochondrial paralogs has occurred in a few post-WGD species (such as *S. pastorianus*, see Supplementary Data Sheet 1 for details).

## Discussion

This study addresses the question of whether paralogous genes with divergent evolutionary origin constituting orthologous pairs, such as the *ScLEU4*/*ScLEU9* vs. *KlLEU4*/*KlLEU4BIS*, follow similar or diverse functional diversification patterns. Previous work established that putative paralogs, arising from segmental or single gene duplications are extant in species of Saccharomycetales upstream the WGD episode (Langkjaer et al., 2003; Dujon et al., 2004). Taking advantage of the MetaPhOrs database^7^ (Pryszcz et al., 2011; Chorostecki et al., 2020), we have estimated that in *K. lactis* 162 gene duplications have occurred since its divergence from the basal species *Yarrowia lipolytica.* Incidentally, some duplicated pairs encode proteins involved in amino acid biosynthesis, such as homologs of the post WGD pairs the *S. cerevisiae SER3*/*SER33* and *LYS20/LYS21*. After the α-IPMS duplication event, which originated the *K. lactis KlLEU4/KlLEU4BIS* pair, episodes of loss occurred in some clades, including within the pre-WGD Saccharomyceteacea. Some species lost the cytosolic paralog. In others, through loss of the mitochondrial pre-sequence, the paralog arising from the original duplication reverted to a presumably cytosolic location. In this work we show that synthesis of α-IPMS could occur either in the cytosol or the mitochondria. Perhaps the successive changes of localization of the α-IPMS are related to the specific ecology and/or physiology of each species.

*ScLEU4* and *ScLEU9* encoded isozymes, are both mitochondrially located; however, a fraction of *Sc*Leu4 resides in the cytosol and could thus synthesize α-IPM in this location. Accordingly, an *oac1*Δ mutant, which is unable to transport α-IPM from the mitochondria to the cytosol, is able to sustain a braditrophic growth feeding from the leucine synthesized in the cytosol (Marobbio et al., 2008). We have previously shown (López et al., 2015) that *ScLEU4* and *ScLEU9* originated from the WGD event, and have functionally diverged, resulting in three mitochondrial isoforms: the leucine-sensitive homodimeric *Sc*Leu4-*Sc*Leu4, the relatively leucine-resistant homodimeric *Sc*Leu9-*Sc*Leu9, and the heterodimeric *Sc*Leu4-*Sc*Leu9 displaying intermediate leucine sensitivity (López et al., 2015).

Our results indicate that *K. lactis KlLEU4* and *KlEU4BIS*-encoded isozymes, which originated from a more ancient small-scale duplication event (SSD), are both leucine sensitive isozymes which, under the conditions of the present study, play a redundant role, since both genes can complement the wild type phenotype to the *Klleu4*Δ *Klleu4bis*Δ leucine auxotrophic mutant. Divergence has been observed in their subcellular localization, *Kl*Leu4 being mitochondrial and *Kl*Leu4BIS cytosolic. We have asked whether each of the *K. lactis* orthologous pair (*KlLEU4*/*KlLEU4BIS*) could complement the complex phenotype of a *Scleu4*Δ *ScLEU9* mutant and the leucine auxotrophy of the *Scleu4*Δ *Scleu9*Δ double mutant. Our results indicate that either *K. lactis* paralog can complement the leucine auxotrophy. However, the complex *Scleu4*Δ *ScLEU9* phenotype is only complemented by the *KlLEU4* gene which is syntenic with *ScLEU4* and *ScLEU9* and encodes the mitochondrial isoform.

### Is the Function of the *K. lactis KlLEU4* and *KlLEU4BIS* Paralogous Genes Redundant?

The growth phenotype of the double *Klleu4*Δ *Klleu4bis*Δ mutant is not restored to wild type levels by the sole addition of leucine, suggesting the existence of an additional requirement not supplemented by this amino acid. The transcription factor *Sc*Leu3 activating function is positively modulated by α-IPM (Sze et al., 1993; Boer et al., 2005). *Sc*Leu3 ligated to α-IPM activates transcription of various genes involved in branched chain amino acid and glutamate biosynthesis (Hu et al., 1995; Kohlhaw, 2003). Absence of α-IPM production in the *Klleu4*Δ *Klleu4bis*Δ double mutant could alter the presumed transcriptional role carried out by the *KlLEU3* orthologous gene (KLLA0D10593g, NCBI Gene ID 2892795, ORF 52% identity with *ScLEU3*). It is not far-fetched to assume that the *K. lactis* ortholog also requires α-IPM-mediated activation. The partial, rather than complete, supplementation by leucine of the double *Klleu4*Δ *Klleu4bis*Δ mutant, as opposed to that found for the *Scleu4*Δ *Scleu9*Δ strict leucine auxotroph, could be due to an α-IPM requirement, not satisfied by leucine. Although *KlLEU4* and *KlLEU4BIS* seem to play redundant roles; it may be that in some physiological conditions the simultaneous activity of both enzymes could be necessary to attain physiological α-IPM levels.

*K. lactis* is a strict aerobic organism, and its respiratory system is not glucose repressed (Schaffrath and Breunig, 2000), *Klleu4*Δ and *Klleu4bis*Δ single mutants show wild type growth rate on either glucose or ethanol as carbon and energy sources, indicating that each paralogous protein is capable to maintain energy and biomass production in aerobiosis.

The physiological function of the different localization of the first step of the pathway in *S. cerevisiae* and *K. lactis* are not obvious. *KlLEU4* and *KlLEU4BIS* seem to play redundant roles; however, the fact that the encoded enzymes show a differential subcellular localization, which has been maintained in several families of the Saccharomycetales (see Supplementary Material) is somewhat of a paradox. Further studies will have to be carried out in order to determine whether these genes could play non-redundant roles and their relation to α-IPM and leucine biosynthesis.

### The Complex *Scleu4*Δ *ScLEU9* Phenotype Can Be Complemented Only by the *Kl*Leu4 Mitochondrial Isozyme

*KlLEU4* and *KlLEU4BIS* encoding respectively, mitochondrial and cytosolic isoforms, are able to fully complement the leucine auxotrophy displayed by *Scleu4*Δ *Scleu9*Δ double mutant.

We have previously shown that over-expression of *ScLEU9* in a *Scleu4*Δ genetic background, results in slow growth (on either glucose or ethanol as carbon sources) and leucine accumulation. *Sc*Leu9-*Sc*Leu9 resistance to leucine feedback inhibition could impact flux control due to increased activity of the biosynthetic pathway (López et al., 2015). Accordingly, when the *Scleu4*Δ *ScLEU9* mutant is complemented with the *ScLEU4* the heterodimeric leucine-sensitive *Sc*Leu4-*Sc*Leu9 isozyme is formed, thus recovering the physiological feedback regulation, wild type leucine biosynthetic rate and consequently wild type growth. The *Kl*Leu4 mitochondrial isoform is able to only partially complement the growth phenotype of the mutant *Scleu4*Δ *ScLEU9*, on either glucose or ethanol, partially mirroring *Sc*Leu4 activity, while the cytosolic *Kl*Leu4BIS isozyme did not improve *Scleu4*Δ *LEU9* growth phenotype either on glucose or ethanol. Both *K. lactis* paralogs can revert the increased *ScLEU9* expression to wild type levels (“Results” sections, Figure 6C). Thus, *Scleu4*Δ *ScLEU9* growth rate complementation by the *KlLEU4* orthologous gene could not only be due to the *KlLEU4* capacity to counteract the enhanced *ScLEU9* expression in a *Scleu4*Δ background. Additionally, the mitochondrial localization of the *Kl*Leu4 protein could in principle enable the formation of a heterodimeric *Sc*Leu9-*Kl*Leu4 isoform, which may have higher leucine sensitivity than the *Sc*Leu9-*Sc*Leu9 homodimer, thus overcoming decreased feedback regulation. While *Kl*Leu4BIS can prevent enhanced *ScLEU9* expression, its cytosolic localization would not allow the formation of the proposed *Sc*Leu9-*Kl*Leu4BIS leucine sensitive heterodimers.

## Conclusion

It can be concluded that the *KlLEU4* and *KlLEU4BIS* paralogous pair have followed a path of functional diversification resulting in different subcellular localization of the encoded enzymes, which at variance from that seen in *S. cerevisiae*, do not differ in leucine sensitivity thus not affecting differentially α-IPMS biosynthesis feedback regulation.

## Data Availability Statement

All datasets presented in this study are included in the article/Supplementary Material.

## Author Contributions

BA-L, JH-G, ER-O, ME-H, ML, JG, GL, and DM performed the experiments. XE-F performed and designed the experiments and contributed to the manuscript writing. MB designed the experiments and obtained the funding. CS designed the experiments, performed the phylogeny analysis, and wrote the manuscript. LR-R designed the experiments and wrote the manuscript. AG designed the experiments, wrote the manuscript, and obtained the funding. All authors contributed to the article and approved the submitted version.

## Conflict of Interest

The authors declare that the research was conducted in the absence of any commercial or financial relationships that could be construed as a potential conflict of interest.

## Footnotes

1 https://mafft.cbrc.jp/alignment/server/

2 http://phylogeny.lirmm.fr/phylo_cgi/alacarte.cgi

3 http://tree.bio.ed.ac.uk/software/figtree/

4 https://ihg.gsf.de/ihg/mitoprot.html

5 http://gpcr.biocomp.unibo.it/bacello/info.htm

6 http://www.cbs.dtu.dk/services/DeepLoc/

7 http://orthology.phylomedb.org/
