# Supplementary material for: In Kluyveromyces lactis a Pair of Paralogous Isozymes Catalyze the First Committed Step of Leucine Biosynthesis in Either the Mitochondria or the Cytosol
Source: Front Microbiol. 2020 Aug 4;11:1843. doi: 10.3389/fmicb.2020.01843 (PMC7418496; doi:10.3389/fmicb.2020.01843)
Supplement: Supplementary file 1 [file Table_1.DOCX]

**TABLE S1.** Deoxyoligonucleotides used in this study.

| **Name** | **Sequence** | **Application** |
| --- | --- | --- |
| A3 | 5’-GGCCCACGTGAAACAAAATTG-3’ | *Klleu4::kanMX4* Fw deletion module, it comprises 22 bp of the 5’UTR (-676 to -654) of *KlLEU4* sequence. |
| A4 | 5’-TTCAACCCAAAGTGCAATGTAGGTGG-3’ | *Klleu4::kanMX4* Rv deletion module, it comprises 26 bp of the 3’UTR (+2304 to +2230) of *KlLEU4* sequence. |
| A5 | 5’-gtcgacctgagcgtacgACTCAATATGGGGTATGGTTGCCTG GTCGTATCACCTTTG-3’ | *Klleu4::kanMX4* Rv deletion module, it comprises18 bp (lowercase) from the pFA6a*-kanMX4* sequence and 40 bp of the 5’UTR (0 to -40) of *KlLEU4* sequence. |
| A6 | 5’-CAAAGGTGATACGACCAGGCAACCATACCCCATATT GAGTcgtacgctgcaggtcgac-3’ | *Klleu4::kanMX4* Fw deletion module, it comprises 40 bp of the 5’UTR (-40 to 0) of *KlLEU4* sequence and 18 bp (lowercase) from the pFA6a-*kanMX4* sequence. |
| A7 | 5’-TAGATACAGTGCAAGTTTGTGGAACTTTTGAAAAGT ATAAACTCCGCTGAatcgatgaattcgagctcg-3’ | *Klleu4::kanMX4* Rv deletion module, it contains 50 bp of *KlLEU4* 3’UTR (+1830 to + 1850) and 19 bp (lowercase) from the pFA6a-*kanMX4* sequence. |
| A8 | 5’-cgagctcgaattcatcgatTCAGCGGAGTTTATACTTTTCAAAAG TTCCACAAACTTGCACTGTATCTA-3’ | *Klleu4::kanMX4* Fw deletion module, it comprises 19 bp of the 3’UTR (+1830 to +1850) of *KlLEU4* sequence and 50 bp (lowercase) from the pFA6a-*kanMX4* sequence. |
| A9 | 5’-GGTGGAAATGACTTTGTCAGGTCG-3’ | *Klleu4BIS::kanMX4* Fw deletion module, it comprises 24 bp of the *KlLEU4BIS* 5’UTR (-738 to -714). |
| A10 | 5’-gtcgacctgcagcgtacgcTTGCTCTAATGACTCTTTACTTCTCT ATGTGCCTGATGGGTTTTCC-3’ | *Klleu4BIS::kanMX4* Rv deletion module, it comprises 19 bp (lowercase) of the pFA6a-*kanMX4* sequence and 47 bp of the *KlLEU4BIS* 5’UTR (0 to -47). |
| A11 | 5’-GGAAAACCCATCAGGCACATAGAGAAGTAAAGAGTC ATTAGAGCAAGcgtacgctgcaggtcgac-3’ | *Klleu4BIS::kanMX4* Fw deletion module, it comprises 47 bp of the *KlLEU4BIS* 5’UTR (-47 to 0) and 18 bp (lowercase) of the pFA6a-*kanMX4* sequence. |
| A12 | 5’-GAAGAAACATGGGAATGCCTTTGGAAGCTTTTGTTC ATGTTTatcgatgaattcgagctcg-3’ | *Klleu4BIS::kanMX4* Rv deletion module, contains 43 bp corresponding to the 3’UTR (+1848 to +1891) of the *KlLEU4BIS* sequence and 19 bp (lowercase) from the pFA6a-*kanMX4* sequence. |
| A13 | 5’-cgagctcgaattcatcgatAAACATGAACAAAAGCTTCCAAAGG  CATTCCCATGTTTCTTC-3’ | *Klleu4BIS::kanMX4* Fw deletion module, it comprises 42 bp of the *KlLEU4BIS* 3’UTR (+1848 to +1891) and 19 bp (lowercase) of the pFA6a-*kanMX4* sequence. |
| A14 | 5’-CGTTGTTCTGGCCAGAATTTCG-3’ | *Klleu4BIS::kanMX4* Rv deletion module, it comprises 22 bp of the *KlLEU4BIS* 3’UTR (+2508 to +2530). |
| A19 | 5’-AGGGCGAACTAACAATACCAGCTCATCGTGACTCAGC  CTCAGCATCTGCAggtgacggtgctggttta-3’ | *KlLEU4* Fw coding sequence for translational fusion to yECitrine, pKT175 sequence is indicated (lowercase). |
| A20 | 5’-TAGATACAGTGCAAGTTTGTGGAACTTTTGAAAAGTA  TAAACTCCGCTGAtcgatgaattcgagctcg-3’ | *KlLEU4* Rv coding sequence for translational fusion to yECitrine, pKT175 sequence is indicated (lowercase). |
| A21 | 5’-TAAACGGTCTTGATAGGGCAAAGAACTTCACTGTCAA  TTCAGCTGCCAATggtgacggtgctggttta-3’ | *KlLEU4BIS* Fw coding sequence for translational fusion to yECitrine, pKT175 sequence is indicated (lowercase). |
| A22 | 5’-TATATATAGTTCAGATATGATGGCGTTTATTTCCATAC  CGCATGGGTAGAtcgatgaattcgagctcg-3’ | *KlLEU4BIS* Rv coding sequence for translational fusion to yECitrine, pKT175 sequence is indicated (lowercase). |
| A23 | 5’-ATGATATTCAGGAACACCGTTGTGCG-3’ | Fw sequence to amplify 4409 bp *KlLEU4-yECitrine* module starting at *KlLEU4* ORF. |
| A24 | 5’-CGGTGAGTGTGGTGATATTCCTG-3’ | Rv sequence to amplify 4409 bp *KlLEU4-yECitrine* module ending at *KlLEU4* 3’-UTR. |
| A25 | 5’-CTTCTGTTCTCCGCTTTGACACTT-3’ | *ScLEU4* Fw promoter region sequence (-514 to -490) to amplify 514 bp. |
| A26 | 5’-**CGCACAACGGTGTTCCTGAATATCAT**GATTGTTCCT  TGTCTTTTTGTAAGG-3’ | *KlLEU4-yECitrine* Fw (*Sc*-*Kl*) hybrid module, it comprises 26 pb homologous sequence (bold) with the ORF of *KlLEU4-yECitrine* module starting at *KlLEU4* ORF and *ScLEU4* promoter region sequence (-25 to -1). |
| A27 | 5’-**ATCAGGAATATCACCACACTCACCG**GAACTTTTCT  GTATTTCAGGACTTATTCG-3’ | *KlLEU4-yECitrine* Rv (*Sc*-*Kl*) hybrid module, it comprises 25 pb homologous sequence (bold) with the 3’ UTR of *KlLEU4-yECitrine* module and *ScLEU4* 3’ UTR region sequence (+1861 to +1889). |
| A28 | 5’-GCGAGTACTTATTGTCCTTTATGCC-3’ | *ScLEU4* Rv 3’ UTR region sequence (+2145 to +2170) to amplify 310 bp. |
| A29 | 5’-ATGCCATTCTACAAAGATCCTTCAGTG-3’ | Fw sequence to amplify 4468 bp *KlLEU4BIS-yECitrine* module starting at *KlLEU4BIS* ORF. |
| A30 | 5’-GGAATTGAACCTGCAACCCTTCG-3’ | Rv sequence to amplify 4468 bp *KlLEU4BIS-yECitrine* module ending at *KlLEU4BIS* 3’-UTR. |
| A31 | 5’-**CACTGAAGGATCTTTGTAGAATGGCAT**GATTGT  TCCTTGTCTTTTTGTAAGG-3’ | *KlLEU4BIS-yECitrine* Fw (*Sc*-*Kl*) hybrid module, it comprises 27 pb homologous sequence (bold) with the ORF of *KlLEU4BIS-yECitrine* module starting at *KlLEU4BIS* ORF and *ScLEU4* promoter region sequence (-25 to -1). |
| A32 | 5’-**AATCGAAGGGTTGCAGGTTCAATTCC**GAACTTT  TCTGTATTTCAGGACTTATTCG-3’ | *KlLEU4BIS-yECitrine* Rv (*Sc*-*Kl*) hybrid module, it comprises 26 pb homologous sequence (bold) with the 3’ UTR of *KlLEU4BIS-yECitrine* module and *ScLEU4* 3’ UTR region sequence (+1861 to +1889). |
| A33 | 5’-CGCGCGGTCGACAGGAGTCCAGGCTCGCTCTCC-3’ | *KlLEU4* Fw coding sequence and promoter region plus 221 bp of the 3’UTR region (-933 to +2051), underlined sequence indicates *Sal*I restriction site to inserted in PRS416 or YEpKD352 plasmids. |
| A34 | 5’-CGCGCGGAATTCCGGTGAGTGTGGTGATATTCCTG-3’ | *KlLEU4* Rv coding sequence and promoter region plus 221 bp of the 3’UTR region (-1000 to +2051), underlined sequence indicates *Eco*RI restriction site to inserted in pRS416 or YEpKD352 plasmids. |
| A35 | 5’-CGCGCGGTCGACCTCCGACATTGATCTCATCCTTCC-3’ | *KlLEU4BIS* Fw coding sequence and promoter region plus 165 bp of the 3’UTR region (-1000 to +1917), underlined sequence indicates *SalI* restriction site to insert in pRS416 or YEpKD352 plasmids. |
| A36 | 5’-CGCGCGGAATTCGCTTTACCCACGAGCATGGCC-3’ | *KlLEU4BIS* Fw coding sequence and promoter region plus 165 bp of the 3’UTR region (-1000 to +1917), underlined sequence indicates *Eco*RI restriction site to inserted in pRS416 or YEpKD352 plasmids. |
| A37 | 5’-AAGCTCGTAGTTGAACTTTGGGTCTGG-3’ | *Kl18S* Fw sequence to amplify internal loading standard probe 18S from *Kluyveromyces lactis*. |
| A38 | 5’-AAGACTTTGATTTCTCGTAAGGTGCCG-3’ | *Kl18S* Rv sequence to amplify internal loading standard probe 18S from *Kluyveromyces lactis*. |
| A39 | 5’-ATGGTTAAAGAGAGTATTATTGC-3’ | Fw sequence of *ScLEU4* from +1 to +23 used for Northern blot probe |
| A40 | 5’-TTATGCAGAGCCAGATGCCGCAG-3’ | Rv sequence of *ScLEU4* from +1837 to +1860used for Northern blot probe |
| A41 | 5’-ATGGTAAAACATTCGTTCATAGCG -3’ | Fw sequence of *ScLEU9* from +1 to +24 used for Northern blot probe |
| A42 | 5’-TTACTCTGCCAGTAGAACATCCC-3’ | Rv sequence of *ScLEU9* from +1792 to +1815 used for Northern blot probe |
| A43 | 5’-CAGGAACACCGTTGTGCGTTTAG-3’ | Fw sequence of *KlLEU4* from +9 to +31used for Northern blot probe |
| A44 | 5’-GAGCTGGTATTGTTAGTTCGCCC-3’ | Rv sequence of *KlLEU4* from +1779 to +1801used for Northern blot probe |
| A45 | 5’-GGCTCCAAGATGGTTAGCTACC-3’ | Fw sequence of *KlLEU4BIS* from +96 to +117 used for Northern blot probe |
| A46 | 5’-GAATTGACAGTGAAGTTCTTTGCCC-3’ | Rv sequence of *KlLEU4BIS* from +1715 to +1739 used for Northern blot probe |
| A47 | 5’-GTTTTGCCGGTGACGAC-3’ | Fw sequence from of *ACT1* from +368 to +384 used for Northern probe |
| A48 | 5’-CTTTCGGCAATACCTGGG-3’ | Rv sequence of *ACT1* from +1227 to +1244 used for Northern probe |
