# Supplementary material for: In Kluyveromyces lactis a Pair of Paralogous Isozymes Catalyze the First Committed Step of Leucine Biosynthesis in Either the Mitochondria or the Cytosol
Source: Front Microbiol. 2020 Aug 4;11:1843. doi: 10.3389/fmicb.2020.01843 (PMC7418496; doi:10.3389/fmicb.2020.01843)
Supplement: Supplementary file 2 [file Data_Sheet_1.docx]

**Supplementary Data**

**Detailed analysis of the phylogeny of** **α-isopropyl malate synthase in the Saccharomycotina**

α-Isopropyl malate synthase (α-IPMS) is conserved throughout the tree of life (with the notable exception of metazoans), including bacteria and archea. It is almost universally conserved in fungi, with the exception of some parasitic or symbiotic basal species. The homologues present in the dicaria form a monophyletic clade, of different bacterial origin that those extant in the sampled Mucormycotina and Chytridiomycota (Larson and Idnurm, 2010).

In *K. lactis* a pre-WGD species, a specific gene duplication event is extant, leading to the *KlLEU4* and *KlLEU4BIS* paralogous gene pair. The occurrence of a more ancient specific gene duplication event than the above mentioned WGD was predicted in PhylomeDB (<http://phylomedb.org/?q=search_tree&seqid=LEU4>) (Huerta-Cepas et al., 2008; Huerta-Cepas et al., 2014).

***Within the Saccharomycotina an early duplication originated a mitochondrially located isoform***

The availability of a plethora of genomes in the Saccharomycotina prompted us to investigate more thoroughly the extent and origin of this early gene duplication.

We thus constructed a phylogenetic tree of α-IPMSs presumed proteins and investigated the putative intracellular localization of each protein. To date, no completely reliable predictor of mitochondrial localization is extant (Sun and Habermann, 2017). For every putative α-IPMS sequence included in the phylogeny shown in **Supplementary** **Figure S2**, we indicate its predicted localization by two different algorithms (see Materials and Methods). Mitoprot is the standardly used algorithm, while we chose BacelLo among others, as it predicted correctly the localization for the four homologues of *S. cerevisiae* and *K. lactis* (López et al., 2015 and this paper). While writing this article we became aware of DeepLoc, a predictor of subcellular localization using deep neural networks (Almagro Armenteros et al., 2017). We thus re-investigated the predicted localization of each protein represented in **Supplementary** **Figure S2***.* This figure shows a Maximum Likelihood phylogenetic tree in cartoon form (for details see Materials and Methods) of the α-IPMSs homologues extant in publicly available databases. We have included as out-groups, representatives of the Taphrimycotina and Pezizomycotina. A majority of the Saccharomycotina include two paralogues in their genomes which form two clearly separated clades, the putative cytosolic clade (PCC) not highlighted and the putative mitochondrial clade (PMC) highlighted in yellow in **Supplementary Figure S2**. In the basal families ([Trigonopsidaceae](https://www.ncbi.nlm.nih.gov/Taxonomy/Browser/wwwtax.cgi?mode=Undef&id=1540145&lvl=3&lin=f&keep=1&srchmode=1&unlock), [Lipomycetaceae](https://www.ncbi.nlm.nih.gov/Taxonomy/Browser/wwwtax.cgi?mode=Undef&id=29827&lvl=3&lin=f&keep=1&srchmode=1&unlock), [Ascoideaceae](https://www.ncbi.nlm.nih.gov/Taxonomy/Browser/wwwtax.cgi?mode=Undef&id=44277&lvl=3&lin=f&keep=1&srchmode=1&unlock), [Saccharomycopsidaceae](https://www.ncbi.nlm.nih.gov/Taxonomy/Browser/wwwtax.cgi?mode=Undef&id=34366&lvl=3&lin=f&srchmode=1&unlock), [Dipodascaceae](https://www.ncbi.nlm.nih.gov/Taxonomy/Browser/wwwtax.cgi?mode=Undef&id=34353&lvl=3&lin=f&keep=1&srchmode=1&unlock), [Trichomonascaceae](https://www.ncbi.nlm.nih.gov/Taxonomy/Browser/wwwtax.cgi?mode=Undef&id=410830&lvl=3&lin=f&keep=1&srchmode=1&unlock) and also Phaffomycetaceae), only one homologue per species is extant, which is predicted to be cytosolic by MitoProt and BacelLo with only a couple of discrepancies, and homogenously cytosolic by DeepLoc-1.0.

All the Pezizomycotina and Taphrinomycotina species included as out groups have only one homologue, which encode a presumably cytosolic enzyme. There is, however, some experimental evidence for a mitochondrial location of the Leu3 protein of *Schizosaccharomyces pombe* (Matsuyama et. al., 2006).

Three species are particularly interesting, as they pinpoint the possible node of gene duplication and the appearance of the mitochondrially located paralogue. These are *Petrozyma xilosa*, *Nakazawaea wickerhamii* and *Pachysolen tannophilus.* These species have been traditionally classified in different families. However, recent work comprising the genome-wide phylogeny of 332 species of the Saccharomycetales has reclassified species belonging to these three genera in one monophyletic clade (CUG-Ala Clade) (Shen et al., 2018). The topology of both the putative cytosolic and mitochondrial paralogues of these three species is absolutely consistent with the new taxonomy. The position of the CUG-Ala clade suggests that this is the more ancient clade where a duplication of the ancestral gene presumably encoding a cytosolic protein has occurred. This would place the duplication at ≈ 200 My before present (Shen et al., 2018). Work with *S. cerevisiae Sc*Leu4 has gone some way to establish the sequences necessary for mitochondrial import. *Sc*Leu4 has two presumed initiation codons in its N terminus. Translation from the first codon results in a mitochondrial form, while initiation at the second methionine results in a cytosolic form (Beltzer et al., 1986; Beltzer et al.,1988). The paralogues shown as cytosolic in **Supplementary Figure S3** are without exception, shorter in their N terminus than their putatively mitochondrially located paralogues and start with a ML(R/K)(D/N)PS sequence, which corresponds to the second N terminus of *Sc*Leu4 (MLKDPS). The paralogues of the CUG-Ala clade clearly illustrate that putative mitochondrial paralogues have acquired a N-terminal extension which could function as a mitochondrial entry sequence (**Supplementary** **Figure S3**). The proteins in the PMC in **Supplementary Figure S2** are predicted to be mitochondrial by DeepLoc-1.0 with some exceptions discussed below.

***In the Saccharomycetaceae episodes of loss of the cytosolic paralogue have occurred, while in most post-WGD species two mitochondrial paralogues are extant***

Episodes of loss of the putative cytosolic paralogue occurs in the pre-whole genome duplication members of the Saccharomycetaceae. Only species of *Kluyveromyces*, *Zygosaccharomyces* and *Eremothecium gossypii* conserve the putative cytosolic paralogue. This is very clear in the *Lachancea*/*Kluyveromyces* clade. We noticed that the experimentally tractable species *Lachancea kluyveri* (Beck et al., 2008) is not represented in our tree. No orthologues were found with Blastp, however both putative cytosolic and mitochondrial orthologues are found with tBlastN (not shown).

A similar situation is extant in the *Zygosaccharomyces*/*Torula* clade and among species of *Eremothecium.* The phylogeny is consistent with at least three independent losses of the putative cytosolic paralogue among the pre-WGD members of the Saccharomycetaceae. Consequently, no paralogues in the PCC are present in any of the post-WGD species. The mitochondrial *KlLEU4* orthologue of *K. lactis* and of a number of pre-WGD of the Saccharomycetaceae are syntenic with *ScLEU4*/*ScLEU9* of *S. cerevisiae* (Yeast Gene Order Browser; Byrne and Wolfe, 2005). Sequence homogenization, probably resulting from gene conversion, must have occurred in this clade. The two sequences of *Candida glabrata* differ by one residue and a short C-terminal extension. In some post-WGD species only one orthologue is present, including in *Saccharomyces pastorianus*, who is itself a hybrid of *S. cerevisiae* and *S. eubayanus* (Nakao et al., 2009). **Supplementary** **Figure S2** suggests that the *ScLEU4* orthologue was conserved in this species, deriving from *S. eubayanus* while the *ScLEU4* orthologue was lost in *S. boulardii*.

Six species within the PMC score as cytosolic by DeepLoc-1.0. Three of these *Brettanomyces naardenensis* II (Pichiacea), *Lodderomyces elongisporus* II and *Scheffersomyces stipitis* II (both Debaryomycetaceae) have a putative cytosolic paralogue in the PCC. We have compared their sequences with the nearest homologue which scores as mitochondrial in all three algorithms. Both *B*. *naardenensis* II and *Scheffersomyces stipitis* II lost the sequences between the two conserved methionines (see **Supplementary Figure S3**) extant in *Dekkera bruxellensis* II and *Suhomyces tanzawaensis* II respectively, which presumably determine mitochondrial entry. The issue is less straightforward for *L*. *elongisporus,* where only a glutamine present in the pre-sequence of *Candida orthopsilopsis* and *C. parapsilopsis* is substituted by a serine.

*Cyberlindnera fabianii*, *Hanseniaspora opuntiae* and *Wickerhamomyces anomalus* are pre-WGD members of the Saccharomycetaceae where a paralogue in the PCC is absent. *C. fabiani* shows several deletions in the putative mitochondrial pre-sequence compared with *C. jadinii,* while a seven amino acid deletion occurs in the pre-sequence of *W*. *anomalus* compared with *W. ciferri.* While only experimental work could determine the actual intracellular localization of these α-IPMS homologues, it seems clear that at least the proteins that have lost the mitochondrial pre-sequence would localize in the cytosol. Thus *B*. *naardenensis* would include two cytosolic paralogues, while some of the pre-WGD of the Saccharomycetaceae would have reverted to a situation where only the cytosolic isoform is present.

**References:**

Almagro Armenteros, J.J., Sønderby, C.K., Sønderby, S.K., Nielsen, H., Winther, O. (2017). DeepLoc: prediction of protein subcellular localization using deep learning [published correction appears in Bioinformatics. 2017 Sep 19]. *Bioinformatics* 33, 3387‐3395. doi:10.1093/bioinformatics/btx431

Beck, H., Dobritzsch, D., and Piskur, J. (2008) *Saccharomyces kluyveri* as a model organism to study pyrimidine degradation. *FEMS Yeast Res*. 8, 1209-1213. doi:10.1111/j.1567-1364.2008.00442.x

Beltzer, J.P., Chang, L.F., Hinkkanen, A.E., and Kohlhaw, G.B. (1986). Structure of yeast *LEU4*. The 5’ flanking region contains features that predict two modes of control and two productive translation starts. *J. Biol. Chem*. 261, 5160-5167.

Beltzer, J.P., Morris, S.R., and Kohlaw, G. B. (1988). Yeast *LEU4* encodes mitochondrial and nonmitochondrial forms of alpha–isopropylmalate synthase. *J. Biol. Chem*. 263, 368-374.

Byrne, K.P., and Wolfe, K.H. (2005) The Yeast Gene Order Browser: combining curated homology and syntenic context reveals gene fate in polyploidy species. *Genome Res*. 15, 1456-1461. doi:10.1101/gr.3672305

Huerta-Cepas, J., Bueno, A., Dopazo, J., and Gabaldón, T. (2008). Phylome DB: a database for genome-wide collections of gene phylogenies. *Nucleic. Acid. Res*. 36, D491-D496. doi:10.1093/nar/gkm899

Huerta-Cepas J, Capella-Gutiérrez S, Pryszcz LP, Marcet-Houben M, Gabaldón T. (2014). PhylomeDB v4: zooming into the plurality of evolutionary histories of a genome. *Nucleic. Acid. Res*. 42, D897-D902. doi:10.1093/nar/gkt1177

Larson, E.M. and Idnurm, A. (2010). Two origins for the gene encoding alpha-isopropylmalate synthase in fungi. *PLoS One* 5, e11605. doi:10.1371/journal.pone.0011605

López, G., Quezada, H., Duhne, M., González, J., Lezama, M., El-Hafidi, M., et al. (2015). Diversification of Paralogous α-Isopropylmalate Synthases by Modulation of Feedback Control and Hetero-Oligomerization in *Saccharomyces cerevisiae*. *Eukaryot. Cell* 14, 564-577. doi:10.1128/EC.00033-15

Matsuyama, A., Arai, R., Yashiroda, Y., et al. (2006) ORFeome cloning and global analysis of protein localization in the fission yeast *Schizosaccharomyces pombe*. *Nat. Biotechnol*. 24, 841-847. doi:10.1038/nbt1222

Nakao, Y., Kanamori, T., Itoh, T., Kodama, Y., Rainieri, S., Nakamura, N., Shimonaga, T., Hattori, M., and Ashikari, T. (2009). Genome sequence of the larger brewing yeast, an interspecies hybrid. *DNA Res*. 16, 115-129. doi:10.1093/dnares/dsp003

Shen, X.X., Opulente, D.A., Kominek, J., Zhou, X., Steenwyk, J.L., Buh, K.V., et al. (2018). Tempo and mode of genome evolution in the budding yeast subphylum. *Cell* 175, 1533‐1545.e20. doi:10.1016/j.cell.2018.10.023
