# Supplementary material for: In Kluyveromyces lactis a Pair of Paralogous Isozymes Catalyze the First Committed Step of Leucine Biosynthesis in Either the Mitochondria or the Cytosol
Source: Front Microbiol. 2020 Aug 4;11:1843. doi: 10.3389/fmicb.2020.01843 (PMC7418496; doi:10.3389/fmicb.2020.01843)
Supplement: Supplementary file 3 [file Image_1.PDF]

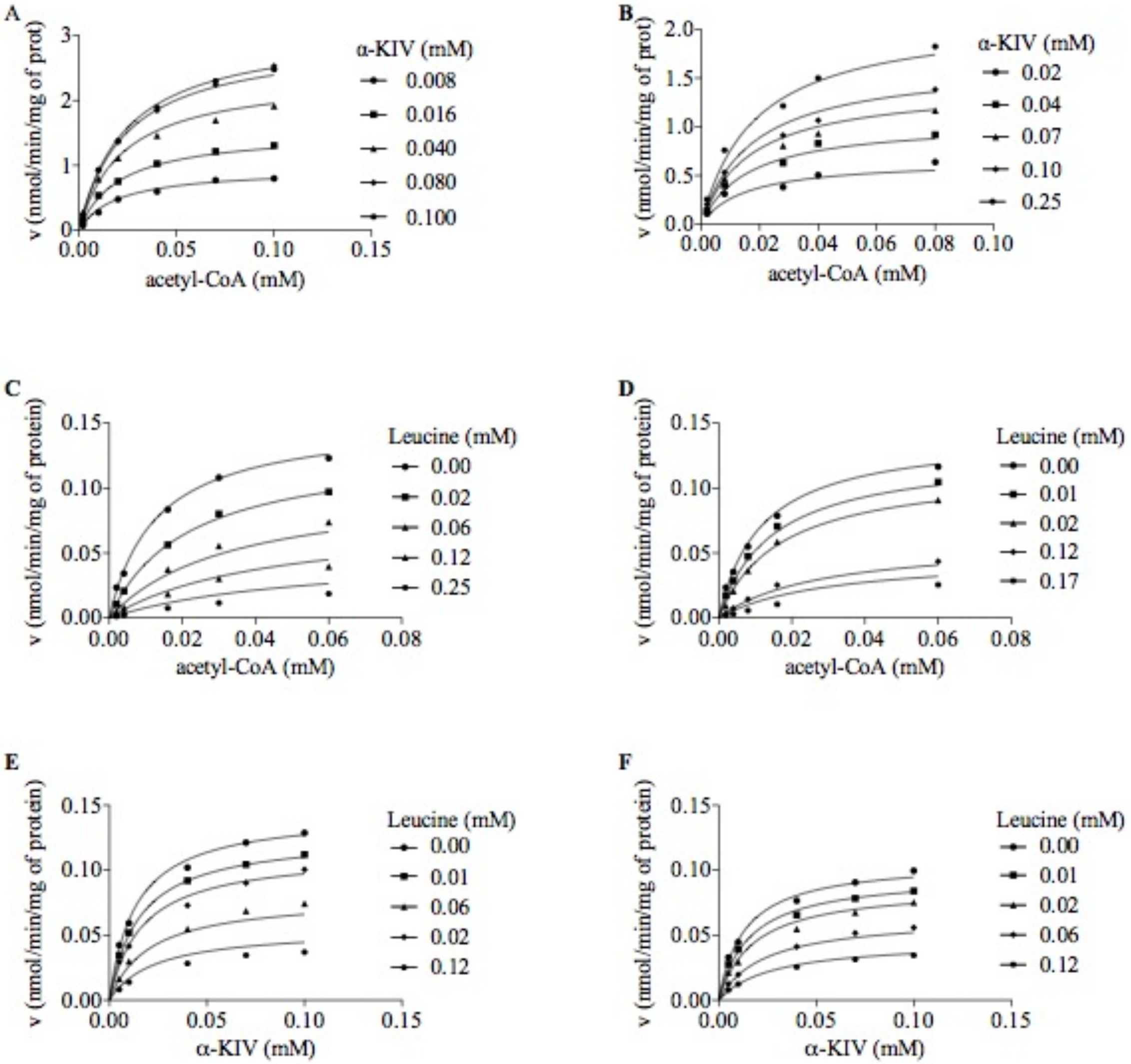

Supplementary Figure S1. *Kl*Leu4 and *Kl*Leu4BIS display similar kinetic behaviour. Saturation curves at different concentrations of a-KIV (top) and leucine (bottom) for *Kl*Leu4BIS (**A, C** and **E**) and KlLeu4 (**B, D** and **F**). Continuos lines represent the global fit to equation 1.
