# Supplementary material for: In Kluyveromyces lactis a Pair of Paralogous Isozymes Catalyze the First Committed Step of Leucine Biosynthesis in Either the Mitochondria or the Cytosol
Source: Front Microbiol. 2020 Aug 4;11:1843. doi: 10.3389/fmicb.2020.01843 (PMC7418496; doi:10.3389/fmicb.2020.01843)
Supplement: Supplementary file 4 [file Image_2.PDF]

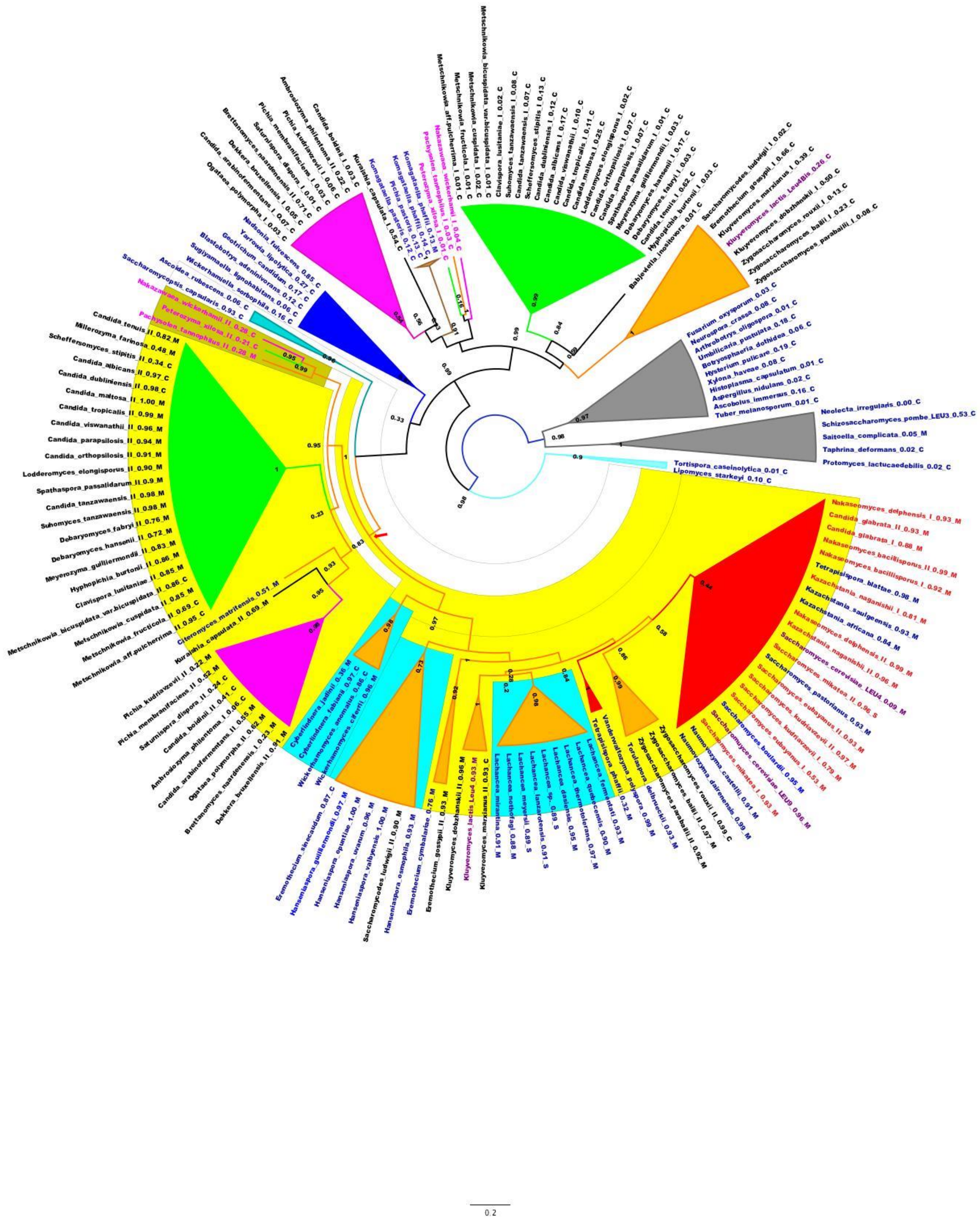
0.2

Supplementary Figure S2. Rooted Maximum Likelihood phylogenetic tree of α-isopropyl malate synthase paralogues of the Saccharomycotina. The tree is shown in a cartoon form. Outgroups are representative species of Taphrinomycotina and Pezizomycotina, shown by two separated grey cartoons. The species of Saccharomycotina are grouped according to standard taxonomy (https://www.ncbi.nlm.nih.gov/taxonomy/).

Light Cyan: Trigonopsidaceae, Lipomycetaceae; Dark cyan: Ascoideaceae, Saccharomycopsidaceae; Blue: Dipodascaceae, Trichomonascaceae. These six families comprise only pre-gene duplication, putatively cytosolic orthologues. Brown: Phaffomycetaceae, this clade also comprises one α-IPMS/species, presumably cytosolic. Green: Metschnikowiaceae, Debaryomycetaceae. Magenta: Picchiceae. Orange: Pre WGD Saccharomycetaceae. Red: Post WGD Saccharomycetaceae. The branches corresponding to the three species of the CUG-Ala clade are colored according to their traditional taxonomy (see text). Some *Incerta sede* sequences are individually indicated with black branches. Highlighted in yellow: Species comprising a mitochondrial paralogue, following a duplication of the ancestral, presumably cytosolic, gene. Highlighted in olive-green, within the clade highlighted in yellow: Mitochondrial paralogues of the CUG-Ala Clade, the outgroup for all species comprising a presumably mitochondrially located paralogue. The red arrow indicates the putative duplication node and origin of the mitochondrial isoform. Highlighted in cyan: Clades where the cytosolic paralogue was subsequently lost. The *Eremothecium* genus includes both species a with a cytosolic and a mitochondrial paralogue and a species with only the mitochondrial isoform.

Species names are shown in full, followed by the probability of mitochondrial location as assessed by Mitoprot and location as predicted by BaceLlo (C, cytosolic, M, Mitochondrial, S, secreted, this was found for four sequences clearly predicted as mitochondrial by Mitoprot). The intracellular localization of all proteins in the tree was re-analyzed using DeepLoc-1.0 (see **Supplementary Data**).

To distinguish the two paralogues in all species comprising these, the species name is arbitrarily followed by I or II, with the exception of the proteins of *S. cerevisiae* and *K. lactis*, where the standard nomenclature is used.

Species names in blue: those where only one paralogue (whether cytosolic or mitochondrial) is present. Species names in black: those where both paralogues in the PCC (Putative Cytosolic Clade) and the PMC (Putative Mitochondrial Clade) are present. Species names in red: post whole genome duplication species where two mitochondrial paralogues are present. Species names in magenta: those belonging to the CUG-Ala Clade, presumably at the root of the duplication and acquisition of a mitochondrial addressing sequence (see also **Supplementary Figure S3**). Species name in purple: the four proteins -*S. cerevisiae* *Sc*Leu9 and *Sc*Leu4 and *K. lactis* *Kl*Leu4 and *Kl*Leu4BIS- concerned by the experimental work (López et al., 2015 and this article). Digits in nodes are Approximate Likelihood ratio test (aLTR). Methodological details are found in the Materials and Methods section and in **Supplementary Data**.
