# Supplementary material for: In Kluyveromyces lactis a Pair of Paralogous Isozymes Catalyze the First Committed Step of Leucine Biosynthesis in Either the Mitochondria or the Cytosol
Source: Front Microbiol. 2020 Aug 4;11:1843. doi: 10.3389/fmicb.2020.01843 (PMC7418496; doi:10.3389/fmicb.2020.01843)
Supplement: Supplementary file 5 [file Image_3.PDF]

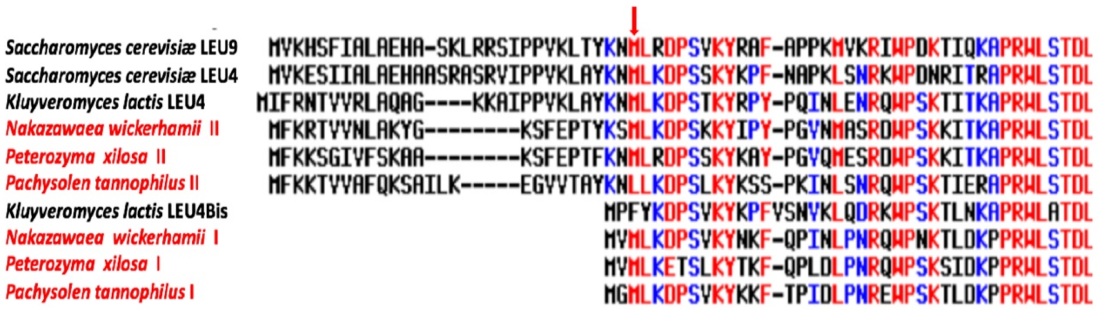

Supplementary Figure S3. Amino-terminus of α-IPMS proteins from representative species. The sequences shown were extracted from a MultAlin alignment (http://multalin.toulouse.inra.fr/multalin/) (Corpet, 1988). The red arrow indicates the highly conserved, alternative initiation methionine of the ScLeu4 protein, which leads to a cytosolic localized isoform (Beltzer et al., 1986; Beltzer et al., 1988). Species in red correspond to species of the CUG-Ala Clade (Shen et al., 2018), the presumed root of the α-IPMS gene duplication in the Saccharomycotina (see **Supplementary Data**, **Supplementary Figure S2** and text). Analysis of these sequences with DeepLoc-1.0 scored the proteins of the CUG-Ala Clade labelled I as cytosolic, and those labelled as II as mitochondrial. The coloring of residues is automatically set by the program and follows the standard Multalin default convention: residues in red, high consensus value; residues in blue, low consensus value; residues in black, no consensus at that position.
